# Analyzing the critical steps in deep learning-based stock forecasting: a literature review

**DOI:** 10.7717/peerj-cs.2312

**Published:** 2024-09-23

**Authors:** Zinnet Duygu Akşehir, Erdal Kılıç

**Affiliations:** Computer Engineering, Ondokuz Mayis University Samsun, Samsun, Turkey

**Keywords:** Deep learning, Stock forecasting, Feature selection, Feature extraction, Denoising, Sliding window, Trading simulation

## Abstract

Stock market or individual stock forecasting poses a significant challenge due to the influence of uncertainty and dynamic conditions in financial markets. Traditional methods, such as fundamental and technical analysis, have been limited in coping with uncertainty. In recent years, this has led to a growing interest in using deep learning-based models for stock prediction. However, the accuracy and reliability of these models depend on correctly implementing a series of critical steps. These steps include data collection and analysis, feature extraction and selection, noise elimination, model selection and architecture determination, choice of training-test approach, and performance evaluation. This study systematically examined deep learning-based stock forecasting models in the literature, investigating the effects of these steps on the model’s forecasting performance. This review focused on the studies between 2020–2024, identifying influential studies by conducting a systematic literature search across three different databases. The identified studies regarding seven critical steps essential for creating successful and reliable prediction models were thoroughly examined. The findings from these examinations were summarized in tables, and the gaps in the literature were detailed. This systematic review not only provides a comprehensive understanding of current studies but also serves as a guide for future research.

## Introduction

Stock forecasting is an interdisciplinary field aiming to forecast the future movements of asset prices in financial markets and represents the intersection between finance and computer science. The predictability of price movements in financial markets is an important topic of discussion within Fama’s random walk hypothesis (Fama, 1995). According to this hypothesis, market prices follow a random walk process, and current prices are independent of past price movements. However, recent research suggests that market prices are predictable in some cases.

In recent years, stock forecasting models have become more complex and data-driven with the advancement of computer technologies such as artificial intelligence, machine learning, deep learning, and big data analytics. These technologies enable the development of models that forecast future price movements by analyzing historical data. However, despite being supported by advanced technologies, stock forecasting inherently involves uncertainties due to the nature of financial markets. The underlying reasons for these uncertainties are company news, economic indicators, political events, investor psychology, *etc*. Consequently, all these factors directly affect the effectiveness of prediction models. Therefore, numerous studies have been conducted on the reliability and accuracy of stock forecasting models, and there are also numerous studies about them.

As a result of the research conducted, it has been determined that creating a successful model for stock forecasting involves some critical steps. These steps include data collection and analysis, feature extraction and selection, noise elimination, model selection, architecture adoption, approach selection for model training, and evaluation of model performance. The correct implementation of each of the steps detailed below plays an important role in creating a forecasting model that can make accurate forecasts and predict the future movements of financial assets. These steps and their details are as follows:

• *Data collection and analysis:* When creating a successful model for stock forecasting, the first step is to collect and analyze data related to the stock item. These data include variables such as stock indices, stock prices, trading volumes, technical indicators, relevant news data, macroeconomic indicators, *etc*. The collection and analysis of these data form the basis of the prediction model and help understand future price movements. Accurate and comprehensive collection of stock market data is a critical factor determining the accuracy and effectiveness of the prediction model.
• *Feature extraction:* Extracting meaningful features from stock data significantly impacts the prediction model’s performance. For example, extracting trends and seasonal patterns from financial time series data. These features can assist the model in predicting future price movements more accurately. Therefore, extracting appropriate features from stock data and utilizing them is vital in creating a successful prediction model.
• *Feature selection:* There are typically numerous features associated with stock data, some of which may be unnecessary or ineffective. Therefore, identifying and removing unnecessary features is important in creating a successful prediction model. Feature selection reduces the complexity of the model and enables better learning by eliminating unnecessary information. Therefore, choosing a suitable feature is an important step for stock forecasting.
• *Noise elimination:* Due to the influence of numerous factors, stock data often contains noise. This noise can adversely affect the performance of prediction models. Therefore, it is important to clean or reduce noise from stock data. This process can be performed using filtering methods, smoothing techniques, outlier detection, *etc*. Noise elimination is an important step in stock prediction because it increases the likelihood of models making more reliable predictions when working with accurate and clean datasets.
• *Model selection and architectural structure:* Selecting an appropriate model and defining its architectural structure are fundamental steps in creating a successful forecasting model for stock prediction. At this stage, the deep learning technique to be utilized should be determined, and their performance on stock data should be evaluated. Additionally, determining the architectural structure of the chosen model is crucial. This architectural structure includes organizing the input and output layers of the model and its internal layers. Establishing the correct architectural structure enables the model to learn better and make more effective predictions on stock data.
• *Selection of training-test approach:* The choice of the training-test approach directly affects the model’s prediction performance, and two primary methods are commonly preferred: the traditional train-test split and the sliding window approach. In the traditional method, a certain percentage of the dataset is used for training, while the remainder is reserved for testing. This method provides a quick and easy performance evaluation but may limit the model’s generalization ability and may not sufficiently account for changing market conditions. On the other hand, in the sliding window approach, training and test sets are created by sliding over a specified period. This method often yields more realistic results as it helps the model adapt to changing market conditions.
• *Performance evaluation:* Evaluating the performance of the constructed prediction model is critical to understanding and improving its effectiveness. At this stage, the defined model architecture should undergo training and validation processes, and the model’s predictive ability should be measured. During this process, the differences between the values predicted by the model and the actual values are observed, and the model’s performance is evaluated using various evaluation metrics such as statistical metrics and trading simulation.

This study conducted a systematic literature review for deep learning-based trend and price prediction of both stock indices and individual stocks. Searches were performed across three different databases using predefined search queries to achieve this. Subsequently, studies were filtered based on exclusion and inclusion criteria applied to these searches. In the final stage, the identified studies were analyzed in detail based on the abovementioned criteria.

### Motivation and contributions

Based on the literature review conducted for this study, it was noted that there is a lack of systematic evaluation encompassing all criteria related to data collection and analysis, feature extraction and selection, noise elimination, model selection, architecture adoption, approach selection for model training, and evaluation of model performance for both stock index and individual stock forecasting. To address this gap, review studies focusing on deep learning models were primarily explored. Six studies published in journals with Q1 or Q2 quarterly rankings between 2020 and 2024 were meticulously examined for this purpose. These review studies were evaluated based on the following eight criteria, and the results of this evaluation are given in Table 1.

**Table 1:** Evaluation of review studies.

**Review studies**	**C1**	**C2**	**C3**	**C4**	**C5**	**C6**	**C7**	**C8**
Stock market forecasting using deep learning and technical analysis: A systematic review (Li & Bastos, 2020)	✓	X	✓	X	X	X	✓	✓
Stock market movement forecast: A systematic review (Bustos & Pomares-Quimbaya, 2020)	✓	X	✓	X	X	X	✓	✓
Decision fusion for stock market prediction: A systematic review (Zhang, Sjarif & Ibrahim, 2022)	✓	X	✓	X	X	X	✓	✓
An overview of machine learning, deep learning, and reinforcement learning-based techniques in quantitative finance: Recent progress and challenges (Sahu, Mokhade & Bokde, 2023)	X	X	✓	X	X	X	✓	✓
News-based intelligent prediction of financial markets using text mining and machine learning: A systematic literature review (Ashtiani & Raahemi, 2023)	✓	X	✓	X	X	X	✓	✓
A systematic literature survey on recent trends in stock market prediction (Balasubramanian et al., 2024)	X	X	✓	X	X	X	✓	✓
This study	✓	✓	✓	✓	✓	✓	✓	✓

• *C1:* How was the search process carried out through databases for the studies to be examined in the review study? Has statistical information been provided about them? Therefore, is the review study systematic?
• *C2:* Have the studies under consideration been summarized individually, with the suggested prediction models in these studies expressed in general terms, and has there been discussion about their contributions to the literature?
• *C3:* Has a detailed investigation been conducted on the input features used in prediction models?
• *C4:* Has information been given about the feature selection or extraction methods used for prediction models?
• *C5:* Has the focus been on proposed approaches to removing noise or denoising in stock data?
• *C6:* Has there been a detailed explanation of the different training-test approaches used in prediction models?
• *C7:* Has information been provided regarding which type of deep learning methods the proposed prediction models in the studies have focused on?
• *C8:* Have the metrics used to evaluate the performance of the proposed models been investigated?

As a result of the reviews, it has been observed that all six review studies focused on the input features and deep learning approaches used in prediction models, along with model performance evaluation. While most of the review studies were systematic, none focused on feature selection/extraction, noise reduction, and train-test approaches. It has been found that only detailed research conducted on input features, prediction methods, and model performance cannot guide more successful stock forecasting models.

Due to the chaotic nature of the data considered for the stock price prediction problem and its high noise level, applying various preprocessing steps is important and necessary. Especially, the successful implementation of these steps may positively affect the model’s overall performance. Also, noise reduction or denoising techniques can enhance the model’s learning accuracy by minimizing unwanted variance in the dataset. Similarly, feature selection or extraction processes can optimize learning by filtering out irrelevant information and focusing on the most relevant features.

Another important point is which of the traditional training-test split or sliding window approach will be used in the training phase of the model. It is important for the dataset to be appropriately divided and for the data used during the model training stage to reflect real-world conditions, as this directly impacts the model’s performance. Consequently, focusing solely on input features and prediction methods is insufficient for creating a successful stock prediction model. Applying appropriate preprocessing steps tailored to the data and using suitable evaluation techniques to improve model accuracy is necessary to obtain more reliable and effective predictions.

As a result of the literature research, it was noted that a systematic review study focusing on all criteria related to data collection and analysis, feature extraction and selection, noise elimination, model selection, architecture adoption, approach selection for model training, and evaluation of model performance for creating a successful deep learning-based stock forecasting model had not been conducted. This situation constitutes our main motivation. To address this gap in the literature, this study conducted a systematic review of deep learning-based stock prediction studies between 2020 and 2024, evaluating each study based on the eight criteria listed above. Therefore, this constitutes the main contribution of this study. Additionally, we believe that this study will shed light on researchers working on stock forecasting problems.

### The audience it is intended for

The intended readership of this study comprises scholars, researchers, and professionals engaged in the interdisciplinary domains of finance, computational finance, machine learning, and artificial intelligence. Primarily, this research targets academics and researchers interested in applying deep learning methodologies in financial time series analysis and forecasting. Furthermore, practitioners involved in quantitative finance, algorithmic trading, and investment management will find the insights gleaned from this study invaluable for refining their predictive modeling strategies. Additionally, graduate students and postdoctoral researchers conducting research in financial data analysis and predictive modeling may benefit from the comprehensive review and analysis presented herein. In summary, this study addresses a scholarly audience interested in advancing the understanding and application of deep learning approaches in financial forecasting.

### Organization

The rest of the study is organized as follows: In the ‘Research Methodology’ section, the selection process for the studies examined in the review is detailed. The ‘Obtained Findings and Results’ section begins with a detailed summary of these studies, followed by a comprehensive examination within the scope of the identified research questions. The ‘Conclusion and Recommendations’ section provides a general evaluation of the examined studies and offers recommendations for future research.

## Research Methodology

This systematic review study begins with a process of defining research questions, focusing on the identified objectives. Then, database scans are performed to select studies that will provide the necessary information to answer these research questions. These scans are performed on the query sentences determined based on predefined criteria. Finally, the examination and elimination process of the studies obtained from the database searches is carried out. This process includes the evaluation of the suitability of the studies to the determined criteria and their contribution to the review objectives. Thus, in-depth research is provided by systematically evaluating the information in the literature, and the basis for future research is created.

### Research strategy

This systematic review study aims to achieve the identified goal by addressing the following research questions:

• *RQ1:* Which deep learning methods are preferred in stock prediction models?
• *RQ2:* Which input features do the proposed prediction models focus on?
• *RQ3:* How often are feature extraction-selection methods used in prediction models, and what is their impact on model performance?
• *RQ4:* What methods are proposed for denoising stock data, and how often are they preferred?
• *RQ5:* Which approach, sliding window or traditional train-test splitting, is more commonly preferred in prediction models?
• *RQ6:* What metrics are used to evaluate the performance of models?

**Figure 1:**
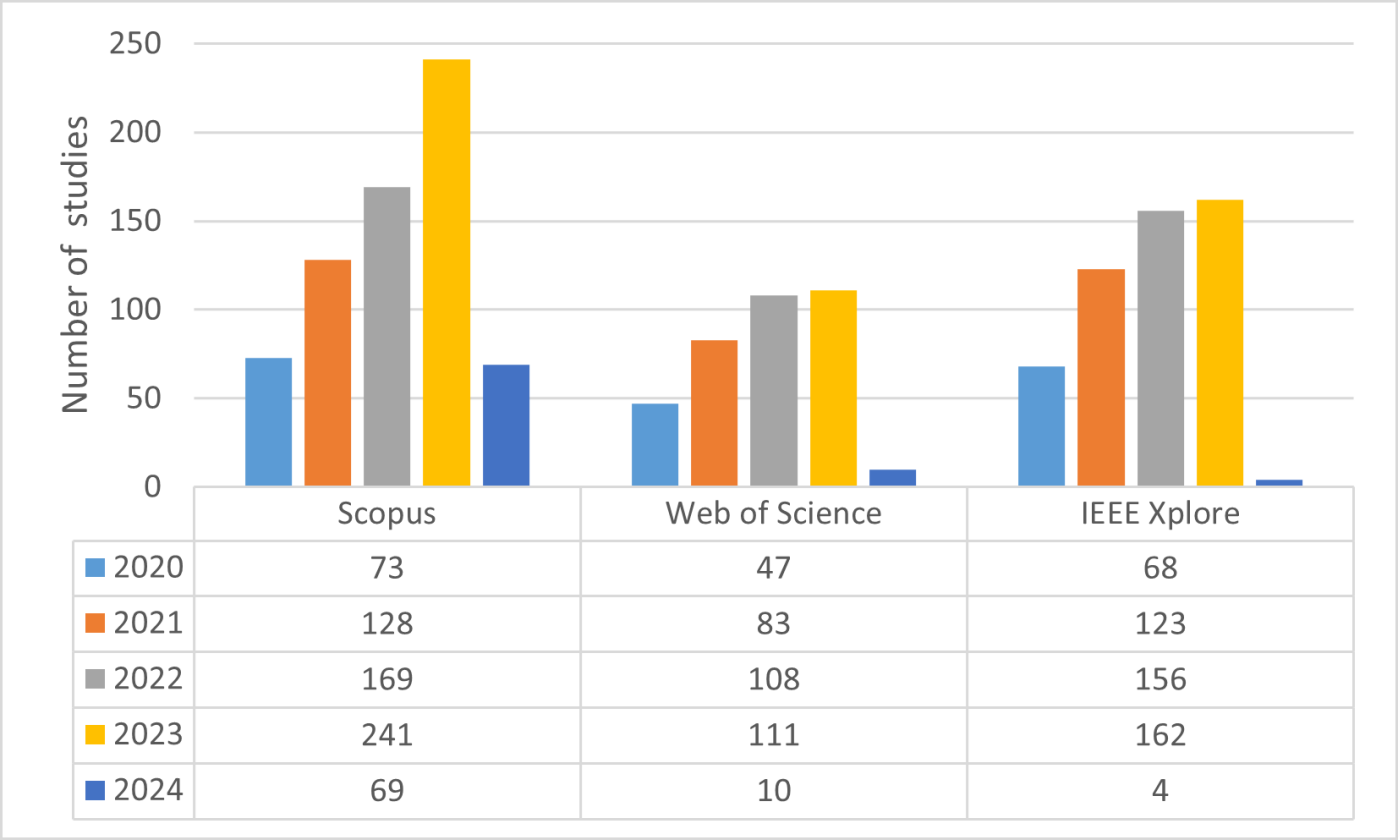
Number of filtered studies with query sentences in databases.

After determining the research questions, literature searches were conducted on four different databases: IEEE Xplore, Scopus, Web of Science, and Google Scholar. The search space was expanded using the “AND” operator for different keywords and the “OR” operator for synonyms of keywords. The query sentences used in this process and information about where they were searched are summarized in Table 2. Based on the information in the table, queries were conducted on the four databases. It has been determined that effective filtering cannot be performed on Google Scholar because queries on this database yield very general results as well as targeted studies. Therefore, Google Scholar searches were disregarded. As a result, the number of studies obtained from searches conducted on three different databases from 2020 to the present is summarized in Fig. 1, and a total of 1,552 studies were filtered through these searches.

**Table 2:** Query statement.

**Database**	**Query statement**
IEEE Xplore	((“stock” OR “financial time serie” OR “financial”) AND (“forecast” OR “predict” )) AND (“deep learning” OR “DL”)
Scopus	(TITLE-ABS-KEY (((“stock” OR “financial time serie” OR “financial”) AND (“forecast” OR “predict” )) AND (“deep learning” OR “DL”)))
Web of Science	AB = (((“stock” OR “financial time serie” OR “financial”) AND (“forecast” OR “predict”)) AND (“deep learning” OR “DL”)))

### Study selection

Some of the 1,552 studies obtained through the queries were found to be searched in multiple databases. Therefore, duplicates were removed to ensure that studies searched in databases were listed only once. In addition, the criteria for elimination and selection for determining whether the listed studies are worth examining within the scope of the review study were defined as follows:

• *Selection Criteria*
  – Studies published between 2020 and 2024
  – Studies published in Q1(top 25% journals with the highest impact factor) or Q2 (journals in the 25–50% impact factor range) quarterly computer science journals
  – Studies focusing on either stock index or individual stock forecasting
  – Studies written in English
• *Elimination Criteria*
  – Studies not focusing on deep learning methods for stock forecasting
  – Studies being either review articles or conference papers
  – Studies focusing on exchange rate forecasting, portfolio optimization, gold price forecasting, financial risk assessment, *etc*.

**Figure 2:**
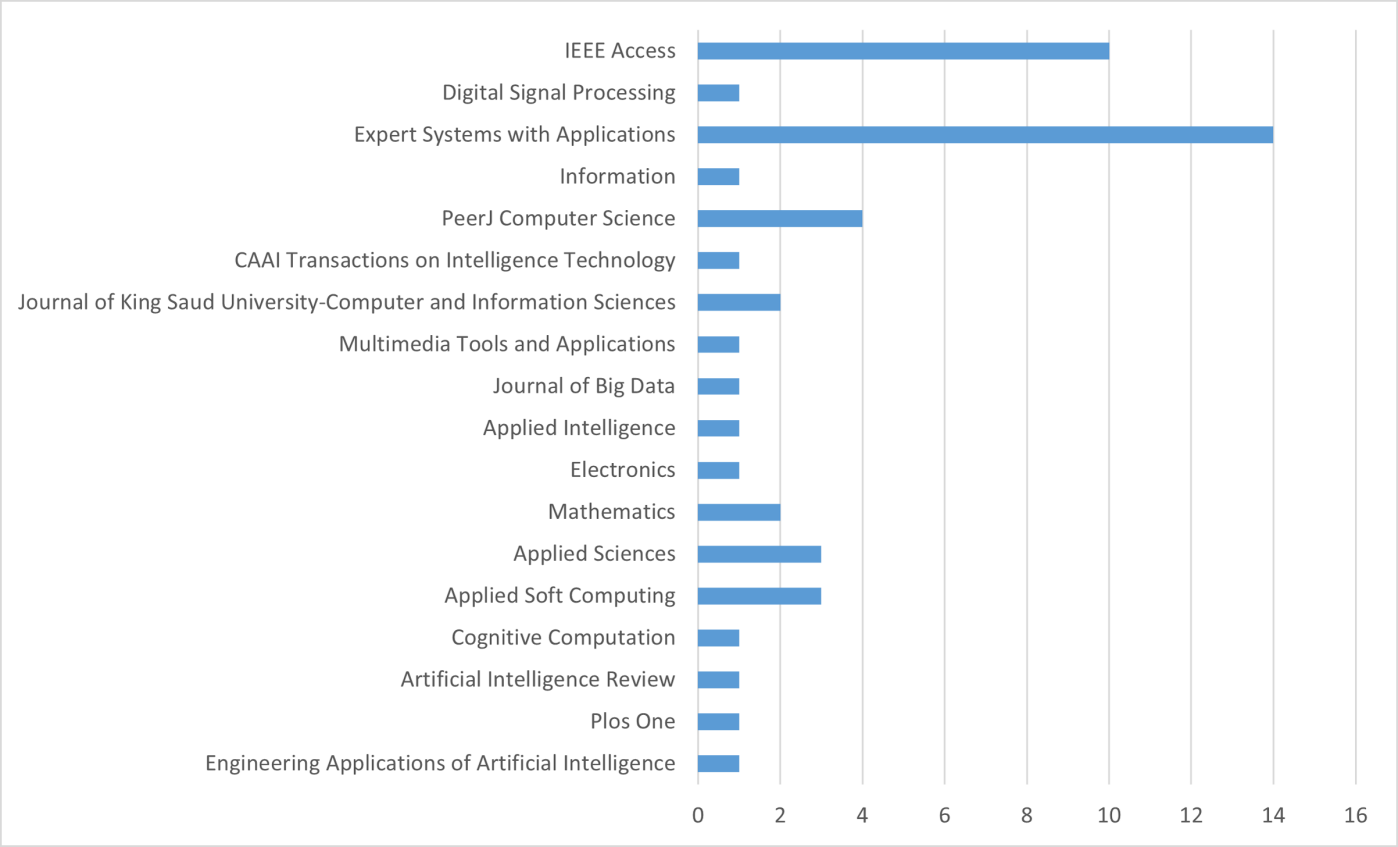
Ranking of journals.

The titles and abstracts of the studies were read, and out of the 1,552 studies identified based on the criteria mentioned above, 49 have been selected and listed in Table 3. These studies include 25 open-access articles. Additionally, an analysis of the journals in which these selected studies were published is provided in Fig. 2. According to this analysis, it is observed that the majority of the selected studies were published in Expert Systems With Applications and IEEE Access journals.

**Table 3:** Studies selected for review.

**Title of paper**	Publication year	Open access
Stock Prediction Based on Genetic Algorithm Feature Selection and Long Short-Term Memory Neural Network (Chen & Zhou, 2020)	2020	✓
Forecasting Stock Prices Using a Hybrid Deep Learning Model Integrating Attention Mechanism, Multi-Layer Perceptron, and Bidirectional Long-Short Term Memory Neural Network (Chen, Zhang & Lou, 2020)	2020	✓
An Efficient Word Embedding and Deep Learning Based Model to Forecast the Direction of Stock Exchange Market Using Twitter and Financial News Sites: A Case of Istanbul Stock Exchange (BIST 100) (Kilimci & Duvar, 2020)	2020	✓
An Improved Deep Learning Model for Predicting Stock Market Price Time Series (Liu & Long, 2020)	2020	X
Stock Trend Prediction using Candlestick Charting and Ensemble Machine Learning Techniques with A Novelty Feature Engineering Scheme (Lin et al., 2021)	2021	✓
A Novel Improved Particle Swarm Optimization With Long-Short Term Memory Hybrid Model for Stock Indices Forecast (Ji, Liew & Yang, 2021)	2021	✓
Combining Deep Learning and Multiresolution Analysis for Stock Market Forecasting (Althelaya, Mohammed & El-Alfy, 2021)	2021	✓
Forecasting Stock Market Indices Using Padding-Based Fourier Transform Denoising and Time Series Deep Learning Models (Song, Baek & Kim, 2021)	2021	✓
Stock Market Index Prediction Based on Reservoir Computing Models (Wang et al., 2021)	2021	X
Predicting Stock Movements: Using Multiresolution Wavelet Reconstruction and Deep Learning in Neural Networks (Peng, Chen & Li, 2021)	2021	✓
Explainable Stock Prices Prediction From Financial News Articles Using Sentiment Analysis (Gite et al., 2021)	2021	X
LSTM-Based Sentiment Analysis for Stock Price Forecast (Ko & Chang, 2021)	2021	✓
Stock Values Predictions Using Deep Learning Based Hybrid Models (Yadav, Yadav & Saini, 2022)	2022	✓
Knowledge Graph and Deep Learning Combined With a Stock Price Prediction Network Focusing on Related Stocks and Mutation Points (Tao et al., 2022)	2022	✓
Stock Market Index Prediction Using Deep Transformer Model (Wang et al., 2022)	2022	X
Modal Decomposition-Based Hybrid Model for Stock Index Prediction (Lv et al., 2022)	2022	X
A Stock Market Trading Framework Based on Deep Learning Architectures (Shah et al., 2022)	2022	X
Implementation of Long Short- Term Memory and Gated Recurrent Units on Grouped Time- Series Data to Predict Stock Prices Accurately (Lawi, Mesra & Amir, 2022)	2022	✓
China’s Commercial Bank Stock Price Prediction Using a Novel K-means-LSTM Hybrid Approach (Chen, Wu & Wu, 2022)	2022	X
AEI-DNET: A Novel DenseNet Model with an Autoencoder for the Stock Market Predictions Using Stock Technical Indicators (Albahli et al., 2022)	2022	✓
StockNet—GRU Based Stock Index Prediction (Gupta, Bhattacharjee & Bishnu, 2022)	2022	X
Multi-Model Generative Adversarial Network Hybrid Prediction Algorithm (MMGAN-HPA) for Stock Market Prices Prediction (Polamuri, Srinivas & Mohan, 2022)	2022	✓
How to Handle Data Imbalance and Feature Selection Problems in CNN-based Stock Price Forecasting (Akşehir & Kiliç, 2022)	2022	✓
Instance-Based Deep Transfer Learning with Attention for Stock Movement Prediction (He, Siu & Si, 2023)	2023	X
Integrating Piecewise Linear Representation and Deep Learning for Trading Signals Forecasting (Chen & Zhu, 2023)	2023	✓
Stock Price Prediction Model Based on Investor Sentiment and Optimized Deep Learning (Mu et al., 2023)	2023	✓
Stock Price Prediction Using CNN-BiLSTM-Attention Model (Zhang, Ye & Lai, 2023)	2023	✓
Shortlisting Machine Learning-Based Stock Trading Recommendations Using Candlestick Pattern Recognition (Cagliero, Fior & Garza, 2023)	2023	X
Combining CNN and Grad-CAM for Profitability and Explainability of Investment Strategy: Application to The KOSPI 200 Futures (Kim et al., 2023)	2023	X
GRU Neural Network Based on CEEMDAN–Wavelet for Stock Price Prediction (Qi, Ren & Su, 2023)	2023	✓
Stock Index Forecasting Based on Multivariate Empirical Mode Decomposition and Temporal Convolutional Networks (Yao, Zhang & Zhao, 2023)	2023	X
Deep Learning-based Integrated Framework for Stock Price Movement Prediction (Zhao & Yang, 2023)	2023	X
Predicting Saudi Stock Market Index by Using Multivariate Time Series Based on Deep Learning (Jarrah & Derbali, 2023)	2023	✓
A Multi- Factor Two- Stage Deep Integration Model for Stock Price Prediction Based on Intelligent Optimization and Feature Clustering (Wang & Zhu, 2023a)	2023	X
Novel Optimization Approach for Stock Price Forecasting Using Multi-Layered Sequential LSTM (Md et al., 2023)	2023	X
A Multi-Parameter Forecasting for Stock Time Series Data Using LSTM and Deep Learning Model (Zaheer et al., 2023)	2023	✓
A Novel Stock Index Direction Prediction Based on Dual Classifier Coupling and Investor Sentiment Analysis (Wang & Zhu, 2023b)	2023	X
A Comparative Study on Effect of News Sentiment on Stock Price Prediction with Deep Learning Architecture (Dahal et al., 2023)	2023	✓
Novel Optimization Approach for Realized Volatility Forecast of Stock Price Index Based on Deep Reinforcement Learning Model (Yu et al., 2023)	2023	X
An Improved DenseNet Model for Prediction of Stock Market Using Stock Technical Indicators (Albahli et al., 2023)	2023	X
Movement Forecasting of Financial Time Series Based on Adaptive LSTM-BN Network (Fang et al., 2023)	2023	X
Forecasting Stock Market Indices Using the Recurrent Neural Network Based Hybrid Models- CNN-LSTM, GRU-CNN, and Ensemble Models (Song & Choi, 2023)	2023	✓
McVCsB: A New Hybrid Deep Learning Network for Stock Index Prediction (Cui et al., 2023)	2023	X
Neural Network Systems With an Integrated Coefficient of Variation-Based Feature Selection for Stock Price and Trend Prediction (Chaudhari & Thakkar, 2023)	2023	X
Forecasting Multistep Daily Stock Prices for Long-Term Investment Decisions: A Study of Deep Learning Models on Global Indices (Beniwal, Singh & Kumar, 2024)	2024	X
An Enhanced Interval-Valued Decomposition Integration Model for Stock Price Prediction Based on Comprehensive Feature Extraction and Optimized Deep Learning (Wang, Liu & Jiang, 2024)	2024	X
A New Denoising Approach Based on Mode Decomposition Applied to The Stock Market Time Series: 2LE-CEEMDAN (Akşehir & Kılıç, 2024b)	2024	✓
Multi Level Perspectives in Stock Price Forecasting: ICE2DE-MDL (Akşehir & Kılıç, 2024a)	2024	✓
A Novel Hierarchical Feature Selection with Local Shuffling and Models Reweighting for Stock Price Forecasting (An et al., 2024)	2024	X

## Obtained Findings and Results

Each of the 49 studies is first summarized in detail in this section, and the prediction models proposed in the studies are explained. This allows researchers to have comprehensive information about each study, compare studies, and fill the gaps in the literature. Following that, the studies have been individually analyzed for each research question.

### Study summaries

Chen & Zhou (2020) pointed out in their study that many factors affect stock prices and emphasized that the factors influencing each stock may vary. They suggested that instead of considering all factors in stock prediction models, focusing on the most important factors influencing each stock is necessary. In this context, they proposed a long short term memory (LSTM)-based forecasting framework that utilizes genetic algorithm (GA) to determine and optimize multiple factor combinations for predicting stock prices. The proposed GA-LSTM prediction model considered 40 factors consisting of technical indicators and financial factors affecting stocks, and GA was used to obtain the importance ranking of these factors. Accordingly, five feature sets were created, including top 30, 20, 10, five, and all factors. The LSTM model was trained using these feature sets, and the optimal factor combination was determined according to the findings. The proposed GA-LSTM prediction model was applied to the China Construction Bank and CSI 300 stock datasets. When the experimental results were evaluated, it was seen that higher prediction performance was achieved with feature sets consisting of the top 10 factors for the China construction bank dataset and the top 20 factors for the CSI 300 stock dataset. Therefore, this study underscores the importance of GA for multi-factor modeling in the stock market and demonstrates its positive impact on more accurate prediction of stock prices.

Chen, Zhang & Lou (2020) proposed a novel hybrid prediction model consisting of an attention mechanism, multi-layer perceptron (MLP), and bidirectional LSTM (BiLSTM) for stock index forecasting. For this proposed model, data from four different sources were combined, including price data from stock indices, technical indicators, historical data from the Google index, and prices of natural resources such as gold, silver, and oil. Accordingly, a total of 45 features were obtained for each stock index. Before moving on to the training phase, feature selection and dimension reduction were performed on these features. Initially, Pearson correlation analysis was conducted on the identified 27 technical indicators, selecting the most important 14 technical indicators. Subsequently, dimension reduction using principal component analysis (PCA) was performed on 31 features. Therefore, as a result of all these processes, the raw data containing four different data sets was transformed into a knowledge base. Then, this dataset was fed into a network consisting of MLP, BiLSTM, and attention mechanisms for predicting the closing values of stock indices. In this network architecture, MLP was used for fast convergence of the algorithm, BiLSTM for extracting temporal features of index data, and the attention mechanism to focus more on crucial temporal information of the neural network. The proposed model was used to predict the closing values of the S&P 500, Russell 2000, NASDAQ, and Dow Jones stock indices, and its prediction performance was compared with seven different prediction models. Experimental results indicated that the proposed model outperformed other prediction models, particularly emphasizing the significant success of the attention mechanism in stock forecasting.

Kilimci & Duvar (2020) focused on financial sentiment analysis using Turkish datasets, unlike recent studies aimed at predicting the stock market’s direction. Their analysis was based on nine high-volume bank stocks from the Istanbul Stock Exchange (BIST 100). They proposed a prediction model based on word embedding and deep learning techniques. They stated that while English news texts have been used for stock market direction prediction, combining deep learning techniques and word embedding methods has yet to be used to predict the direction of Turkish stocks and markets. Accordingly, individual comments and news texts from various sources such as Twitter, KAP, Mynet Finans, and Bigpara were collected. After collecting and cleaning the datasets, nine prediction models were proposed by combining Word2Vec, GloVe, and FastTex word embedding techniques with recurrent neural network (RNN), LSTM, and convolutional neural network (CNN) deep learning approaches to predict the direction of BIST 100. When evaluating the classification performance of the proposed models, it was observed that the combination of FastText and LSTM generally made successful predictions, and RNN and LSTM methods outperformed CNN in all datasets. Therefore, this study shows that combining word embedding techniques and deep learning models provides an important analytical tool for investors.

Liu & Long (2020) proposed a hybrid forecasting framework for daily closing price prediction of stock market indices. The proposed EWT-dpLSTM-PSO-ORELM prediction model combined empirical wavelet transform (EWT) based decomposition, dropout strategy, particle swarm optimization (PSO) based LSTM deep network optimization, and outlier robust extreme learning machine (ORELM) based error correction methods. Three different datasets were used to demonstrate the proposed model’s effectiveness: the S&P 500 Index, China Minsheng Bank (CMSB), and the Dow Jones Industrial Average (DJI). Approximately three years of historical price data were collected for these data sets, and the first two years of this data were used in the model’s training, and the remaining one year was used in the testing. To evaluate the prediction performance of the proposed model, it was compared with three different prediction models from the literature using traditional backpropagation neural network (BPNN), random forest (RF), and single LSTM methods. Additionally, to investigate the impact of each module in the proposed model on prediction performance, a total of seven models were created: dpLSTM, dpLSTM-PSO, EWT-dpLSTM-PSO, dpLSTM-PSO-ORELM, dpLSTM-PSO-RELM, dpLSTM-PSO-WRELM, and dpLSTM-PSO-ELM. All these prediction models were applied to each dataset, and the closing prices of stock indices for the next day were predicted. The comparisons showed that the EWT-dpLSTM-PSO-ORELM model outperformed the BPNN, RF, and single LSTM-based prediction models for all three datasets. Therefore, it was proven that the proposed hybrid model achieved more accurate prediction results than single models based on machine learning or deep learning methods. Furthermore, comparisons with the other seven models revealed that applying the EWT algorithm resulted in the decomposition of complex stock closing prices into stable sub-layers, and the combination of ORELM error correction with EWT decomposition methods significantly improved prediction accuracy. The experiments also showed that the integrated decomposition and error correction model had higher prediction accuracy. At the same time, the dropout strategy and PSO algorithm enhanced the prediction performance of the LSTM network. Thus, the experimental results demonstrated that the proposed EWT-dpLSTM-PSO-ORELM prediction model outperformed other deep learning methods or single models in terms of prediction accuracy. Additionally, it was expressed as future work that this proposed prediction framework would be applied to other financial time series predictions.

Lin et al. (2021) combined traditional candlestick chart analysis with machine learning methods such as logistic regression, support vector machine (SVM), K-nearest neighbors (KNN), RF, gradient boosting decision tree (GBDT), and LSTM for stock prediction. In the study, an eight-trigram classification scheme was developed in feature engineering, and the importance of various technical indicators was thoroughly examined for short-term stock prediction. Additionally, an innovative feature engineering scheme compatible with a machine learning method selection framework was proposed to automatically select the most suitable machine learning method for different candlestick charting patterns. The proposed prediction framework was applied to all stocks in the China stock market. Accordingly, daily stock data for 18 years (between 2000 and 2017) were collected. Then, a feature set was created for each trading day, including the date, intra-day pattern, inter-day pattern, 21 technical indicators, and the next day’s closing price. To examine the effect of technical indicators in the proposed forecast model, these indicators were discussed under four groups: overlap, momentum, volume, and volatility. Thus, six different feature sets, including without indicators, with all indicators, volume indicators, overlap indicators, momentum indicators, and volatility indicators, were applied with six machine learning methods. When the results obtained were evaluated, it was observed that RF and GBDT generally had a good forecasting ability in short-term forecasts, but LSTM performed worse than other methods in this scenario. It has also been stated that using fewer momentum and volatility indicators in short-term forecasts increases forecast performance to a satisfactory level. Finally, an investment strategy based on forecasting frameworks was developed, and experimental results confirmed that this strategy could provide significant economic returns on individual stocks and portfolios.

Ji, Liew & Yang (2021) proposed a hybrid model consisting of improved particle swarm optimization (IPSO) and LSTM for predicting stock market indices. It has been stated that when the standard PSO algorithm is used for hyperparameter optimization of the LSTM network, rapid convergence to the local optimum occurs, negatively affecting the prediction performance. In response to this issue, they proposed the IPSO method based on a new inertia weight and adaptive mutation factor for more effective hyperparameter optimization. The performance of the proposed model was evaluated using four different performance metrics on five stock market indices: Australian Stock Market (ASM), DJI, IXIC, Hang Seng Index (HSI), and Nikkei 225. For the experiments, 10-year daily historical data of stock market indices were used. Additionally, the performance of the proposed IPSO-LSTM model was compared with support vector regression (SVR), LSTM, and PSO-LSTM models. The comparative analysis demonstrates that the proposed IPSO-LSTM model performs best and exhibits successful generalization ability across different market indices.

Althelaya, Mohammed & El-Alfy (2021) proposed an innovative financial time series prediction approach, combining multiresolution analysis and the LSTM. The study investigated the effect of stationary wavelet transform (SWT) and EWT multiresolution analysis methods on predicting the S&P 500 index. To this end, approximately eight years of data from January 1, 2010, to February 20, 2018, for both long-term (30 days ahead) and short-term (next day) predictions of the S&P 500 index were collected from Yahoo Finance. The proposed hybrid model consists of three stages: in the first stage, financial time series data was decomposed using multiresolution analysis. Then, a separate LSTM network was trained for each resolution level. Finally, the final target value was estimated by combining the obtained estimates. Experimental results demonstrate that EWT outperformed SWT for both long-term and short-term predictions of the S&P 500. The results indicate that better patterns were obtained with EWT, suggesting the potential for creating more effective prediction models.

Song, Baek & Kim (2021) highlighted that noise in financial time series data impedes the learning of prediction models and causes time delays. To address this issue, they proposed a padding-based Fourier transformation denoising approach (P-FTD) to eliminate noise and resolve the data delay problem when reverting the time series to its original form. Hybrid prediction models, including P-FTD_RNN, P-FTD_LSTM, and P-FTD_GRU, which combine deep learning methods with P-FTD. These models were designed to predict the closing prices of S&P 500, SSE, and KOSPI stock indices. The performance of the proposed models was compared with basic gated recurrent unit (GRU), LSTM, and RNN models that did not utilize the denoising approach. Additionally, the effectiveness of the P-FTD approach was validated by comparing it with other denoising methods used in stock prediction models in the literature. Experimental results demonstrated that the hybrid models based on P-FTD outperformed the basic models in terms of prediction accuracy and also reduced time delays more effectively compared to other denoising methods.

Wang et al. (2021) emphasized the importance of using innovative deep-learning methods to predict price movements in financial markets. They researched the applicability of the reservoir computing (RC) method for predicting the daily closing price of stock indices. RC, a specialized type of RNN model, differs from traditional deep learning methods in that it only trains the weights of the output layer instead of all weights, significantly simplifying the training process. The primary motivation of Wang et al. (2021) in this study was the successful application of RC in time series prediction problems due to its nonlinear structure. However, it has not yet been applied to financial time series. To this end, RC prediction models were developed using three different network structures: random network, small-world network, and scale-free network. The proposed model predicted the closing price of seven stock indices, specifically S&P 500, NYSE, DJI, NASDAQ, FTSE 100, Nikkei 225, SSE for the next day. The proposed model’s prediction performance was evaluated in terms of statistical metrics (mean squared error (MSE), mean absolute error (MAE), *R*^2^, root mean square error (RMSE), mean absolute percentage error (MAPE)) and compared with four different predictions, including LSTM and RNN. The results indicated that the RC model generally exhibited competitive performance compared to leading models like LSTM and RNN, demonstrating its potential as a successful alternative for stock index prediction. However, some limitations of the model were also highlighted. Firstly, the study only covered seven stock indices, which could hinder the generalization of prediction models. Secondly, the dataset used only included daily closing prices, and the model’s performance was not evaluated on shorter time intervals (*e.g.*, 1 h, 1 min). Lastly, the proposed model was not evaluated based on trading strategies developed for real-world applications. These limitations were identified as potential research areas for the broader evaluation and development of the RC model.

Peng, Chen & Li (2021) stated the significance of predicting the direction of stock prices in financial markets, highlighting it as a complex and challenging problem. To overcome this problem, they proposed combining wavelet analysis and deep learning methods to develop a prediction model. In the developed model, deep neural network (DNN) was chosen as the deep learning method, and db4 was selected as the wavelet function. To evaluate the effectiveness of the proposed model, medium-term (11 days) stock movement prediction was conducted using stock data from 168 US companies. Additionally, the performance of the proposed model was compared with machine learning methods such as Bayesian, RF, and artificial neural networks (ANN). The results indicated that this prediction model achieved an average accuracy rate of 75%, with particularly high accuracy rates achieved for some stocks such as Alibaba Group Holding and Toyota Motor. Comparative analyses showed that the deep learning method provided more stable and reliable predictions than other machine learning algorithms. Therefore, the primary contribution of this study was emphasized to be the effectiveness of deep learning and wavelet analysis methods in medium-term stock direction prediction. It has been stated that future studies aim to delve deeper into trading strategies and further examine the impact of financial indicators in detail.

Gite et al. (2021) emphasized the complexity of the stock market influenced by positive and negative sentiments in media news and proposed an effective model for predicting stock opening prices. This proposed model combines sentiment analysis based on news headlines with deep learning methods, incorporating the interpretability power of explainable artificial intelligence (XAI). Financial news headlines were collected from Pulse, allowing compilation of financial news, while Indian Stock Markets price data was obtained from Yahoo Finance. Two different models were developed for predicting stock opening prices. In the first model, only price data (open, high, low, close) was used as input, and LSTM was employed as the method. In the second model, sentiment analysis was performed on news headlines to determine whether each headline had a negative or positive sentiment. Then, this headline data was combined with price data and fed as input to the LSTM-CNN model. When evaluating the performance of the proposed two models, it was observed that the LSTM model achieved an accuracy of 88.73%, and the LSTM-CNN model achieved the same accuracy of 74.76%. Consequently, it was determined that the combination of LSTM and XAI provided higher accuracy than other models. It is stated that in future studies, research will be conducted on automatic predictions from news headlines on financial websites.

Ko & Chang (2021) proposed a hybrid model based on sentiment analysis and deep learning methods to predict the opening price of six stocks on the Taiwan Stock Exchange. They used three different categories of data for their proposed model. The first category consisted of daily transaction data of stocks, including information on opening, closing, high and low prices, and volume. The other two categories were textual data from news and forum posts. For the proposed model, first of all, sentiment analysis was performed separately on news and forum post data using the bidirectional encoder representations from transformers (BERT) method. This sentiment analysis obtained the textual data’s class (positive or negative) and probability information. Then, a total of four features were obtained for each type of data. A dataset was created using nine features obtained from textual and historical price data, and an LSTM model was trained on this dataset. Additionally, three different feature sets were created, including the feature set of historical price data, the feature set of news, and the feature set of forum posts. The experimental results showed that the sentiment analysis features of news or forum posts improved the model’s prediction performance. The LSTMNF model, which utilized all three data types, exhibited superior performance compared to the other three models. These results demonstrate the significant role of emotions in news and forums in the stock market. The authors also suggested that considering different types of sentiments would positively contribute to the model’s prediction performance and expressed their intention to conduct further studies.

Yadav, Yadav & Saini (2022) proposed two different deep learning-based models aimed at achieving more accurate and faster prediction of high-frequency and rapidly fluctuating stock prices. In the first proposed model, the FastRNN (Kusupati et al., 2018) method based on the ResNet architecture developed by Microsoft in 2018 was used to predict stock prices quickly. The aim was to achieve reasonable prediction accuracy with lower computational costs by leveraging FastRNN. In the second model, in addition to FastRNN, CNN and BiLSTM methods were employed to enhance prediction accuracy further. This model aimed to capitalize on the advantages of FastRNN to achieve higher prediction accuracy. The proposed models were applied to minute data of Facebook, Uber, Apple, and Nike stocks traded on the New York Stock Exchange. The performance of the models was evaluated in terms of both execution time and RMSE error metric and compared with four basic prediction models such as autoregresive integrated moving average (ARIMA), LSTM, FBProphet and CNN-LSTM. Experimental results demonstrated that the proposed models improved prediction accuracy and enhanced computational speed compared to other prediction models. This suggests that the proposed models could benefit investors, especially in livestock price prediction.

Tao et al. (2022) stated that numerous factors influence the stock market, yet many studies in the literature do not consider the interactions between stocks and sudden change points. In this direction, they proposed a deep learning-based hybrid model focusing on the relationship between stocks and change points. This proposed hybrid model consists of three sub-networks, each obtaining different features related to the stock. Subsequently, these features are merged to predict the stock’s closing price. The details of these sub-networks are provided as follows:

• In the first sub-network, the historical price data of the stock is given as input to the ConvLSTM model, which is obtained by combining the LSTM model with the convolution structure, and the features are obtained.
• The second sub-network utilizes the same architecture as the first one, but the input data differs. Here, a knowledge graph is used to explore the relationship between stocks, and based on this graph, a market information vector is obtained. This vector is then fed into the ConvLSTM model.
• In the third sub-network, sudden changes in stock prices are considered. First, sudden change points are identified, and then a weight matrix is created based on the distance of each trading day to these change points. The motivation behind creating such a matrix is that “the shorter the distance, the greater the impact of the change point”. This matrix data is fed into the graph convolutional network (GCN) model to extract features.

Additionally, the model faces significant errors during training due to these sudden change points in stock price data. To address this issue, a piecewise loss function was proposed. This proposed hybrid forecasting framework was applied to four stocks in the Chinese market. Experimental results showed that the proposed model outperformed all other models for all four stocks, with the ConvLSTM model providing the closest prediction to the proposed model.

Wang et al. (2022) investigated the effectiveness of the transformer model, one of the deep learning methods, in forecasting stock market indices. Several important factors explain why they chose the transformer model as their focus. Firstly, the transformer model has achieved significant success in natural language processing, demonstrating its effectiveness in processing textual data. Secondly, the multi-head attention mechanism of the transformer is effective in emphasizing specific features and identifying important relationships, which allows for better modeling of complex relationships in stock markets. A third important factor is that the transformer model is relatively new in stock market prediction, indicating its potential for achieving more successful forecasts. The proposed transformer model was used to predict the closing values of the S&P 500, HSI, and Nikkei indices, and its performance was compared with traditional deep learning methods such as RNN, LSTM, and CNN regarding returns and statistical metrics. The experiments revealed that the transformer outperformed other models in predicting the closing prices of stock market indices. Moreover, when evaluating the models’ performances in terms of returns, similar results were obtained, indicating that the transformer model yielded higher returns. However, it was noted that this study had various limitations, such as focusing on a single stock market index and not considering the relationships among global financial markets. Future research should overcome these limitations and explore the applications of the transformer in other financial markets.

Lv et al. (2022) highlighted the inadequacy of traditional forecasting models in predicting stock market indices, particularly noting that the noise in financial time series adversely affects the model’s predictive performance. In this regard, they conducted detailed research on deep learning methods to successfully predict the closing prices of stock market indices, proposing a hybrid prediction model based on CEEMDAN-DAE-LSTM. This proposed model addresses the complexity and noise of stock market index data and consists of three stages. Firstly, the complete ensemble empirical mode decomposition with adaptive noise (CEEMDAN) approach was applied to the closing price data of stock market indices to decompose the data into a series of intrinsic mode functions (IMFs), resulting in a less noisy and more stable representation. In the second stage, the deep autoencoder (DAE) method was used to extract deep features and remove irrelevant information, while in the final stage, the prediction process was performed using the LSTM method, a deep learning approach. To evaluate the prediction performance of the CEEMDAN-DAE-LSTM model, six datasets consisting of the closing prices of stock market indices, including the Shanghai Composite Index (SH), Shenzhen Stock Exchange Index (SZ), HSI, Nikkei 225, DJI, and S&P 500, were utilized. Additionally, the performance of the proposed model was compared with five different prediction models, namely Single LSTM, Single RNN, EMD-DAE-LSTM, CEEMDAN-PCA-RNN, and CEEMDAN-PCA-LSTM. Experimental results demonstrate that the proposed model outperforms other models on these various stock market indices, improving prediction accuracy. The findings indicate that the CEEMDAN-DAE-LSTM model combines the advantages of CEEMDAN, DAE, and LSTM methods to provide a more effective prediction capability. To guide future research, it is suggested that macroeconomic variables and factors such as news sentiment be added to the dataset to enhance diversity and individual improvements for each proposed module to improve the model’s prediction capability.

Shah et al. (2022) proposed a deep learning-based hybrid model consisting of CNN and LSTM methods to predict the closing price of the Nifty 50 stock market index. For the feature set of the proposed model, in addition to the raw price data of the index, technical indicators, foreign indices, exchange rates, and commodity price data were utilized, resulting in a total of 48 features. The CNN method was employed to extract high-level features from these obtained features, while the LSTM method was applied to predict the next-day value of the Nifty 50 index. The proposed model achieved a MAPE rate of 2.54% on the training dataset, representing a significant improvement compared to similar studies. Additionally, the proposed CNN-LSTM model was compared with the traditional buy-and-hold strategy. With the proposed model, a return of 342% was achieved in 10 years for the Nifty 50 index, while the traditional buy-and-hold strategy yielded a return of only 107%. This indicates the superiority of the CNN-LSTM model in terms of returns compared to the traditional buy-and-hold strategy. It is noted that future research will focus on conducting more detailed investigations to better adapt the prediction model to noise and random events.

In their study, Lawi, Mesra & Amir (2022) created and evaluated eight new architectural models to predict the stock prices of AMZN, GOOGL, BLL, and QCOM. These models combined the LSTM and GRU models with a four-neural network block architecture to identify joint movement patterns in the stock market using time series data. Therefore, four different LSTM/GRU model architectures for stock price prediction were proposed: the direct model, downsizing model, tuned downsizing model, and stabilized downsizing model. These models used differently processed output shapes to predict stock prices. The proposed eight models were evaluated using three accuracy measures, namely MAPE, root mean squared prediction error (RMSPE), and rooted mean dimensional percentage error (RMDPE), and upper, average, and lower accuracy values were obtained for each. The findings showed that GRU called direct model (Model-1) provided the highest training and test data accuracy.

Based on literature reviews conducted by Chen, Wu & Wu (2022), it has been observed that satisfactory prediction performance has been achieved with deep learning-based models in the scope of stock prediction. However, it is noteworthy that most of the proposed models in these studies used data from a single stock. Additionally, it has been observed that the lack of consideration for the influence of similar stocks within the same group negatively affects the long-term prediction performance. Based on the findings of these studies, a new hybrid model based on deep learning was proposed to improve prediction performance. In the proposed KD-LSTM model, 16 bank stocks with similar trends listed on the Chinese stock market were clustered using the k-means method with dynamic time warping (DTW) distance metric. The value of k was set between 2 and 5 during clustering, and based on the clustering results, the data of four bank stocks identified to belong to the same cluster each time were used to train the LSTM model. Experimental results reported that the clustering approach used in the prediction model positively affected the prediction performance.

Gupta, Bhattacharjee & Bishnu (2022) proposed a new model called StockNet, based on a GRU, to predict the CNX Nifty index’s opening price and overcome the overfitting problem. They introduced a novel data augmentation approach with their proposed model. The StockNet model consists of two parallel modules: an injection module to address the overfitting issue and an investigation module for predicting the index value. Due to the limited amount of data available for the proposed model, overfitting became a challenge in training deep-learning models with such limited data. To mitigate this issue, they proposed a GRU-based data augmentation approach. During the data augmentation process, they considered ten stocks highly correlated with the stock index and explored combinations of these ten stocks in the injection module. To evaluate the performance of the proposed StockNet model, both the real dataset of the CNX Nifty 50 index and the synthetic dataset were used, and comparisons were made with six different prediction models, including DNN and RNN. The comparisons concluded that StockNet exhibited the best performance among the six models.

Polamuri, Srinivas & Mohan (2022) noted the increasing significance of generative adversarial networks (GAN) in recent years, particularly in computer vision. However, they observed that GAN was not widely used for stock market prediction due to the challenging process of hyperparameter tuning. Taking this as their primary motivation, Polamuri, Srinivas & Mohan (2022) proposed a hybrid forecasting framework utilizing GAN to perform stock market prediction. In the proposed model, LSTM and CNN models were used for the two essential components of GAN, namely the generator and the discriminator, respectively. Additionally, reinforcement learning and Bayesian optimization were employed to overcome the difficulty of hyperparameter tuning in the GAN model. The proposed hybrid model also incorporates feature extraction, feature selection, and dimensionality reduction approaches for data preprocessing. An autoencoder was utilized for feature extraction from stock market data, Extreme Gradient Boosting (XGBoost) was employed for filtering features based on their importance, and PCA was used for dimensionality reduction. After the data preprocessing step, the prediction process was carried out using the GAN model. While the proposed model achieved satisfactory performance, it is noteworthy that the computational cost of the model was quite high.

Akşehir & Kiliç (2022) have noted through their literature review that the CNN model has been successfully applied in stock prediction problems. However, they observed data imbalance problems in studies where stocks’ next-day trade action (buy/sell/hold) was predicted using a 2D-CNN model. They emphasized that the labeling approach was the main factor in this problem. In this regard, they proposed a new rule-based labeling approach to address the data imbalance problem. To investigate the impact of this proposed labeling approach on model performance, they predicted the next-day trade actions of stocks in the Dow30 index. For this purpose, the historical price data of stocks were obtained from Yahoo Finance, and each trading day was labeled using the proposed labeling approach. Subsequently, input variables that could positively influence the model’s prediction performance were determined. The most commonly used 15 technical indicators in the literature, along with external factors such as gold and oil prices, were considered for this purpose. Additionally, a correlation-based feature selection method was applied to these variables. Six different input variable sets were created based on both the feature selection approach and the information obtained from the literature review. These input variable sets were transformed into images to train the 2D-CNN model. Both traditional and sliding window training-test approaches were applied during the model training process, and the model’s performance was compared with other CNN-based prediction models in the literature. Experimental results demonstrated that the proposed labeling approach effectively addressed the data imbalance problem. Furthermore, it was observed that the CNN prediction model with the proposed feature selection and labeling methods achieved a 3–22% higher accuracy rate than other CNN-based models in previous studies.

He, Siu & Si (2023) noted that deep learning models are commonly used for stock direction prediction, but the performance of these models could be negatively affected, especially for newly publicly offered stocks, due to the scarcity of historical price data. To address this issue, they proposed an example-based deep transfer learning model (IDTLA) equipped with an attention mechanism. The proposed model utilized a deep transfer learning approach to find effective ways of learning from limited training data. For stock direction prediction, new instances were first created using the IDTLA architecture, and then the entire dataset was trained with the LSTM model.

Chen & Zhu (2023) pointed out that while various artificial intelligence algorithms are used to create prediction models for financial market forecasting, evaluating these models often focuses on accurate price prediction. They focused on determining buy-sell points to achieve high and consistent profits and proposed an effective method. This proposed method consists of piecewise linear representation (PLR), CNN, and LSTM approaches, named PLR-CNN-LSTM. Data from various stocks belonging to companies in Turkey and the United States were considered to evaluate the performance of the PLR-CNN-LSTM model, and historical price data for these stocks were gathered. Subsequently, 15 technical indicator values were calculated using this price data, and a dataset was created. After dataset creation, the PLR method was used to label buy/sell/hold points and identify turning points. Then, considering period values ranging from 3 to 23 for each technical indicator, two-dimensional image representations were created to obtain 20x15 images. These images were fed into the CNN-LSTM model to predict future stock buy-sell signals. Comparisons were made with the PLR-CNN-TA and PLR-LSTM models to evaluate the performance of the proposed PLR-CNN-LSTM model. The comparisons revealed that the proposed PLR-CNN-LSTM model made more successful predictions, resulting in higher profits. However, the main problem here is that after labeling, there is more hold-labeled data than buy/sell-labeled data, making it difficult to differentiate between buy-sell signals. Therefore, this situation also affects the performance of the prediction model.

Mu et al. (2023) combined two data types influencing stock closing prices and proposed the MS-SSA-LSTM hybrid model for predicting these prices. In this proposed hybrid model, comments related to stocks were first collected, and sentiment analysis was performed to obtain a sentiment score. Then, the historical price data of stocks and sentiment index were combined and fed into an LSTM model optimized with the sparrow search algorithm (SSA). The proposed MS-SSA-LSTM model was applied to six different stocks in the Chinese market. Additionally, six other models were created to investigate the effect of each module, including multiple data sources and SSA-optimized LSTM, on the model. Experimental results showed that the MS-SSA-LSTM model made closer predictions to the stock closing price. Additionally, it was observed that using multi-source data and SSA for hyperparameter tuning positively influenced the prediction performance of the stock prediction model.

Zhang, Ye & Lai (2023) stated that various prediction methods ranging from traditional time series techniques to machine learning-based models have been proposed for stock index prediction, achieving a certain level of accuracy with these prediction models. In this direction, they proposed the CNN-BiLSTM-attention hybrid model to improve the accuracy of stock index prediction. In this model, CNN was used to extract temporal features of time series data, followed by BiLSTM to learn dynamic change patterns from these features. Additionally, an attention mechanism was applied to the extracted features to automatically assign weights, thereby reducing the influence of irrelevant information and exploring deeper temporal correlations. Subsequently, the closing value of the stock index was predicted using a dense layer. The proposed CNN-BiLSTM-attention model was first applied to predict the closing value of the CSI 300 index and compared with LSTM, CNN-LSTM, and CNN-LSTM-attention prediction models. Experimental results showed that the proposed hybrid model outperformed the other three models, thus making close predictions of the closing value of the CSI 300 index. To confirm the prediction stability and robustness of the model, experiments were conducted on 11 stock indices, including three Chinese and eight international stock indices. The results indicated that the proposed model effectively predicted both Chinese and other countries’ stock indices and possessed a certain degree of generalization. Additionally, Zhang, Ye & Lai (2023) stated that this study relied on stock indices’ price data, but many factors influence stocks or indices. Therefore, it was noted that the proposed model has structural limitations. In this regard, it was suggested that various information collected from different sources related to stock prices be integrated into the prediction model to improve prediction accuracy.

Cagliero, Fior & Garza (2023) highlighted the increasing interest in machine learning-based trading systems utilizing stock market data and information provided by candlestick patterns. However, they pointed out various disadvantages of these developed models. Firstly, machine learning models tend to generate many buy/sell signals, leading to a high false positive rate. Secondly, models trained on datasets created from candlestick formations and price data face a dimensionality problem. This study proposed separating the machine learning and pattern recognition steps to overcome these disadvantages and create a short-term stock trading system. The proposed model primarily focused on various machine learning models, such as MLP, SVM, RF, LSTM, *etc*., to predict the direction of stocks for the next day. A pattern-based filtering process was conducted in the pattern recognition part, considering pattern features such as confidence level and freshness. Then, the proposed pattern recognition approach was used to prioritize the recommendations of machine learning models and verify their reliability. Three different agreement strategies were determined by looking at the suggestions of the two methods. This proposed model was applied to stocks in the S&P 500 and FTSE MIB 40 indices. It was observed that separately addressing machine learning methods and pattern recognition positively influenced the trading strategy.

Kim et al. (2023) conducted a study focusing on predicting the direction of the KOSPI 200 index and identifying the features the model emphasized during this prediction. Additionally, a trading simulation was conducted using the identified features. For the proposed model, firstly, the 30-minute data of the KOSPI 200 index was collected, and direction labeling was performed based on consecutive data. Subsequently, 16 technical indicators were considered, and 16x16 images were obtained by considering 16-period values starting from 7 and increasing by three units up to 52. The CNN model was trained on the generated image dataset. Then, to confirm which technical indicators or features the model focused on during direction prediction, the gradient-weighted class activation mapping (Grad-CAM) method was applied. Trading simulations were conducted using the important features identified by the Grad-CAM method. The proposed trading strategy was compared with various strategies, such as a simple buy-and-hold strategy and a strategy based on the most commonly used buy/sell technical indicators. Experimental results demonstrated that the proposed strategy yielded higher returns compared to others.

Qi, Ren & Su (2023) proposed a hybrid forecasting model for stock index prediction consisting of GRU, CEEMDAN, and wavelet transform. CEEMDAN and wavelet transform methods were used to remove noise from the index data, while GRU was utilized to predict the next day’s index value. The stock index data was first decomposed into IMFs using the CEEMDAN method in the proposed denoising approach. Despite containing some helpful information, the IMF with high-frequency components also contained more noise. Therefore, the noise was removed by applying wavelet transform instead of directly discarding these components. After denoising the stock index data using the CEEMDAN-wavelet transform approach, the GRU model was trained on the denoised data for prediction. To evaluate the performance of the GRU based on the CEEMDAN-wavelet hybrid model, S&P 500 and CSI 300 index data were used, and performance comparisons were made with GRU, LSTM, ARIMA, CNN-BiLSTM, and ANN models. Experimental results demonstrated that the proposed model outperformed traditional models in making predictions. However, challenges such as the influence of human factors on financial markets and the inability to model emotional factors exist. Therefore, future research may consider methods such as news analysis and sentiment analysis to enhance the model’s effectiveness.

Yao, Zhang & Zhao (2023) emphasized the complexity of modeling and predicting stock market indexes, highlighting the inadequacy of existing prediction models in the literature. To overcome this challenge, they proposed a hybrid model called MEMD-TCN based on multivariate empirical mode decomposition (MEMD) and temporal convolutional networks (TCN). In the proposed model, the COHLV stock index data, encompassing closing price, opening price, highest price, lowest price, and trading volume, were initially decomposed into IMFs using MEMD. Subseries were then obtained for each feature. Then, subseries of the same frequency were fed as input to the TCN network. Finally, the predicted results of all subseries were combined to obtain the closing values of the stock indexes for the next day. Data from seven stock market indexes in China, the United States, Europe, and the Asia-Pacific region were used to evaluate the performance of the proposed MEMD-TCN model. Additionally, the model’s prediction performance was compared with 14 models, including traditional econometric, machine learning, and hybrid models. The experiments demonstrated that the proposed MEMD-TCN model outperformed all other benchmark models regarding prediction performance. Moreover, it was observed that combining different decomposition algorithms with the TCN model further improved prediction performance. Furthermore, it was stated that this study provides a general model that can be used not only for stock market indexes but also for other research areas.

Zhao & Yang (2023) conducted a study addressing the challenge of predicting stock price directions due to the characteristics of stock price data. They proposed a new prediction framework called SA-DLSTM by combining sentiment analysis, denoising autoencoder, and LSTM model. For their proposed model, they collected price data related to the HSI index and textual data from various forum websites associated with the index. They first used the denoising autoencoder method to extract features from the price data, obtaining features strongly correlated with price data while effectively removing noise. Then, they performed sentiment analysis on the collected textual data using the emotion-enhanced CNN (ECNN) method to obtain a sentiment index. The features extracted from the stock price and sentiment index data were then fed into an LSTM network for stock price direction prediction. To evaluate the performance of the proposed SA-DLSTM model, it was compared with traditional machine learning and statistical methods. Additionally, trading simulations were conducted to investigate the model’s impact on profitability. The experimental results demonstrated that the SA-DLSTM model achieved the highest prediction accuracy and captured suitable trading points (buy/sell signals), resulting in high returns.

Jarrah & Derbali (2023) proposed a deep learning-based prediction model to predict the weekly closing prices of three stocks in the Saudi Stock Market Index: Mobily, STC, and Zain. Their study preferred the LSTM method from deep learning techniques and compared the performance of both single-variable and multi-variable LSTM prediction models. For the proposed prediction models, they first removed noise from the stock price data using the exponential smoothing (ES) approach. Then, for single-variable analysis, only the closing price data were used, while for multi-variable analysis, the closing, opening, highest, and lowest price data were inputted into the LSTM model to predict the weekly closing values of the stocks. The prediction results of both models were evaluated using metrics such as MAE, MSE, RMSE, and MAPE. The evaluations revealed that the multi-variable model made more accurate predictions than the single-variable model. The results highlight the effectiveness of multi-variable models and the importance of noise reduction approaches in stock price prediction. Furthermore, the authors emphasized the need for future studies to focus on making longer-term predictions and examining the impact of different economic factors.

Wang & Zhu (2023a) identified two main shortcomings in decomposition-integration-based prediction models for stock price prediction after conducting literature research. First, in decomposition-based models, only the closing price of the stock data is usually considered, while other factors influencing the stock are ignored. Second, prediction results are linearly combined after training the separately obtained sub-series resulting from decomposition. To address these shortcomings, they proposed a new prediction model using the idea of adding effective influencing factors and utilizing classification prediction. The proposed multi-factor two-stage deep integration model for predicting stock index closing prices consists of four stages: multi-factor analysis, feature clustering, classification prediction, and nonlinear integration. In the first stage, the most important factors influencing stock prices were determined using the XGBoost method in the multi-factor analysis module, considering both basic factors such as opening price, highest price, lowest price, *etc*., and non-basic factors such as oil price, interest rate, gold price, exchange rate, consumer confidence index, *etc*. Then, the dataset containing these features was divided into train and test datasets. In the second stage, feature clustering was performed using the k-means method on the training dataset, grouping similar data to facilitate the model’s learning process and improve the accuracy of predictions. In the classification prediction stage, an optimized LSTM model with a genetic algorithm was trained separately on each cluster obtained from clustering. Finally, the prediction results obtained in the previous stage were combined nonlinearly in the nonlinear integration module. The proposed model was used to predict the closing prices of the Shanghai Stock Exchange Composite Index, Shenzhen Stock Exchange Component Index, and New York Stock Exchange Composite Index. Additionally, comparisons were made with single LSTM, EMD-LSTM, CEEMDAN-LSTM, and VMD-LSTM models to evaluate the model’s effectiveness. Experimental results showed that the proposed model outperformed the other models in all indices. In future studies, the proposed model will be further examined for stock direction prediction and applied to other fields, such as the oil and exchange markets.

Md et al. (2023) proposed the multi-layer sequential LSTM model for predicting stock prices with high accuracy. The proposed MLS-LSTM model consists of three sequential LSTMs and one dense layer. The MLS-LSTM model consists of three sequential LSTM layers and one dense layer. This prediction model divides the normalized time series data of stock prices into time steps to determine the relationship between the stock’s past and future closing prices. The proposed model was applied to the 5-year closing price data of Samsung stock, and its performance was compared with various machine learning methods such as MLP, SVM, linear regression, and deep learning methods including RNN, single layer LSTM, and CNN in the literature. The experiments showed that the proposed prediction model achieved an average error percentage of approximately two percent and outperformed other models in making accurate predictions. It was noted that the three sequential LSTM layers used in the MLS-LSTM model prevented the occurrence of overfitting and exhibited superior performance compared to single-layer LSTM. In future studies, integrating fundamental and sentiment analysis methods into the prediction process was also aimed to provide a more reliable and effective approach for stock price prediction.

Zaheer et al. (2023) highlighted that predicting stock prices is a challenging problem due to the complex and noisy nature of financial data, despite it being an interesting area for investors and researchers. They conducted a literature review and observed that previous studies generally focused on a single stock parameter such as predicting the closing price. Building upon this observation, Zaheer et al. (2023) aimed to predict the high and closing values of the Shanghai Composite Index using six price data parameters: open, close, high, low, volume, and adjusted close. To accomplish this prediction task, they trained CNN, LSTM, and RNN models each with a single layer in parallel using the same input data. After training, the model with the highest *R*^2^ value was selected. Comparisons were made with two-layer LSTM and RNN models, as well as CNN-RNN and CNN-LSTM models, to evaluate the performance of the proposed hybrid prediction model, totaling five deep learning models. The results indicated that the single-layer RNN model proposed by Zaheer et al. (2023) had the lowest MAE and RMSE compared to other models, with an *R*^2^ value close to one. Among the proposed models, CNN showed the worst performance. Additionally, comparing single-parameter and two-parameter predictions revealed that two-parameter predictions yielded more accurate results. In future studies, they aim to conduct more in-depth research into predicting stock prices. They plan to develop a more comprehensive prediction model by incorporating risk factors and other variables to enhance accuracy.

Wang & Zhu (2023b) focused on predicting stock trends, which play a crucial role in financial decision-making processes. They investigated the impact of textual content created by investors on stock behaviors and observed that such textual content has significantly influenced stock behaviors in recent years. However, they noted that existing studies in the literature often overlook the diminishing effect of sentiment index over time by assigning equal weight to textual content. To address this issue and predict the trend of stock indexes, they proposed a prediction model based on sentiment analysis and dual classifier coupling. Firstly, they developed a sentiment index weighted based on reading volume for the proposed model. Then, considering the diminishing effect over time, they corrected the weighted sentiment index using the exponential weighted moving average model. Finally, they created a dual classifier coupling model by combining CNN and SVM algorithms to predict the direction of the stock index. To evaluate the performance of the proposed prediction model, they used two Chinese stock indexes, SSE 50 and CSI 300. Additionally, they compared the performance with single-model approaches such as MLP, stochastic gradient descent (SGD), linear regression, SVM, LSTM, and CNN, as well as hybrid models like CNN-LSTM, CNN-SGD, and CNN-BiLSTM. The findings indicated that adding the sentiment index could significantly improve the accuracy of stock market trend prediction. Moreover, the proposed CNN-SVM model outperformed other basic and hybrid models in making accurate predictions. They also suggested that a hybrid prediction model consisting of the sentiment index and different classification algorithms could be used in other time series prediction problems.

Dahal et al. (2023) stated the increasing need to create accurate and reliable prediction models in the face of the growing complexity of financial markets. They observed through literature reviews that both singular and hybrid models created using LSTM, GRU, and CNN are widely used in stock price prediction. In their study, they aimed to examine the impact of financial news on stock prediction by utilizing LSTM and GRU to create four different prediction models. Two of these models, LSTM and GRU, were trained solely using price data of the stock index: open, high, low, close, volume, while the other two models, LSTM-News and GRU-News, incorporated financial news data in addition to the price data. To evaluate the performance of the proposed models, approximately 13 years of price data for the S&P 500 index were collected, along with textual data containing at least 25 news items for each day. For the LSTM-News and GRU-News models, sentiment scores were obtained using the VADER tool from the financial news data. These sentiment scores were combined with the price data to train the LSTM-News and GRU-News models. Experimental results indicated that the LSTM-News and GRU-News models outperformed the LSTM and GRU models trained solely using price data. This finding demonstrated the enhancing effect of financial news data on the prediction accuracy of the models.

Yu et al. (2023) proposed a hybrid model called GVMD-Q-DBN-LSTM-GRU to predict stock market indices’ realized volatility (RV) accurately. This proposed model involves the optimization of RV sequences using the gray wolf optimization algorithm and variational mode decomposition (VMD) to decompose them into IMFs. Each IMF is then trained using deep belief network (DBN), LSTM, and GRU deep learning methods, followed by the integration of prediction results by the Q-learning algorithm to determine their weights. This model predicted the RV sequences of the Shanghai Stock Exchange Composite Index, Financial Times Stock Exchange Index, and S&P 500 index using 5-minute data for the selected indices. Ten prediction models comprising both single and hybrid models were employed to evaluate the model’s performance. The compared models included BPNN, extreme learning machine (ELM), SVR, DBN, LSTM, GRU, DBN-LSTM-GRU, GVMD-DBN-LSTM-GRU, Q-DBN-LSTM-GRU, and WT-Q-DBN-LSTM-GRU. The performance of these models was assessed using metrics such as MAE, MSE, heterogeneous mean absolute error (HMAE), and heterogeneous mean squared error (HMSE). The results indicated that the GVMD-Q-DBN-LSTM-GRU model outperformed other models and effectively predicted RV sequences. Therefore, the study’s main contributions include optimizing VMD for decomposing RV sequences, training each IMF with deep learning models, and integrating prediction results with a reinforcement learning algorithm. Future research aims to conduct more comprehensive analysis in the RV prediction domain and address more complex scenarios for multivariate prediction.

Albahli et al. (2023) proposed a prediction model to forecast the future trends of stocks and assist investors in the decision-making process based on this forecast. Historical price data of stocks were obtained for the proposed prediction model, and 18 technical indicator values were calculated from this data. Subsequently, an autoencoder method was applied to reduce the features on the 18 technical indicators to obtain a simpler set of technical indicators. The reduced technical indicator data and the historical price data of stocks were combined and provided as input to the DenseNet-41 model for prediction. The proposed DenseNet-41 model was derived by reducing the number of dense blocks from four to three compared to the traditional DenseNet, effectively addressing the problem of overfitting and obtaining deep features. This prediction framework was applied to 10 stocks in the S&P 500 index. Various machine learning and deep learning methods were compared to evaluate the model’s performance. The comparisons demonstrated the superior performance of the proposed model, with the most significant contribution attributed to the feature extraction capability of the DenseNet-41 model. In another study conducted by Albahli et al. (2022), the same prediction framework was addressed, with the only difference being the use of the 1D DenseNet model instead of the DenseNet-41 model. When comparing the performance of prediction models created using DenseNet-41 and 1D DenseNet methods, it was observed that using the DenseNet-41 method resulted in lower error metrics and, thus, more successful predictions.

Fang et al. (2023) noted that while the LSTM model is one of the most advanced models for predicting the direction of financial time series, it does not perform well in long-term prediction due to these time series’ non-linear and non-stationary characteristics. To overcome this problem, they proposed a modified adaptive LSTM model. The proposed prediction model consists of an LSTM-BN network with a multi-path parallel structure. Unlike a simple LSTM network, the LSTM-BN network combines LSTM layers with batch normalization and dropout layers to prevent overfitting during model training. Additionally, to enhance the stability of the prediction model, they adopted a parallel structure comprising identical multiple LSTM-BN networks. In this parallel structure, a single LSTM-BN network model exists; each model was trained with the same dataset, and a voting method was used for the final prediction result. The only difference among the LSTM-BN models in this parallel structure lies in their initial parameters inherited from the previous model. In other words, the current network parameters of the models were considered as the initial parameters for the next LSTM-BN network model, thereby reducing the computational cost during the fine-tuning process of the parameters. Furthermore, an adaptive-cross entropy loss function was proposed to improve the prediction performance at sharp change points in financial time series. The proposed parallel LSTM-BN prediction model was applied to predict the direction of the S&P 500, CSI 300, and SSE 180 indices and compared with five different prediction models. The comparisons revealed that the proposed prediction model achieved the highest accuracy and provided the highest return.

Song & Choi (2023) utilized three different hybrid models based on RNN, namely CNN-LSTM, GRU-CNN, and ensemble model, to predict the closing values of the DAX, DJI, and S&P 500 stock indices. Daily price data of the stock indices were used for this prediction. Additionally, a new feature called “medium” was proposed by taking the average of the intraday high and low values. Thus, in the proposed models for stock index prediction, six variables such as open, high, low, volume, change and medium were used as input features and different historical observation values of 5-21-42 days were considered. Four different feature sets were created to investigate the proposed feature’s impact on the models’ prediction performance. The experiments revealed that the proposed medium feature and considering a 42-day historical observation period improved the prediction performance of the models. Furthermore, the ensemble model created by placing RNN, GRU, and LSTM models parallelly outperformed the other models.

Cui et al. (2023) highlighted the difficulty in predicting stock index data due to their nonlinear and nonstationary characteristics. Upon reviewing existing studies in the literature, they found that various statistical and machine learning-based methods were used for index prediction. However, due to the noise in index data, obtaining reliable results using a single method in the prediction model was challenging. Therefore, they researched proposed hybrid models for stock index prediction and categorized them into three main categories: pure machine learning/deep learning hybrid model, time series-deep learning hybrid model, and decomposition-deep learning hybrid model or decomposition-reconstruction prediction framework. This study proposed a new hybrid model named McVCsB based on the decomposition-reconstruction prediction framework for stock index prediction. This model comprises multi-channel VMD, convolutional block attention module (CBAM), and BiLSTM methods. VMD was used to denoise stock index data, CBAM was used to extract deep features, and the BiLSTM model was used to predict the stock index with the obtained features. To evaluate the performance of the proposed model, various experiments were conducted on SSCI, STI, FTSE, and S&P 500 indices, and different prediction models were created to assess the impact of each component of the hybrid model on model performance. The comparisons revealed that the proposed decomposition-reconstruction prediction framework was the most successful prediction model and that each model component positively impacted prediction performance. Additionally, it was noted that the choice of decomposition algorithm influenced the performance of the proposed model and that macroeconomic factors were not considered in this study.

Chaudhari & Thakkar (2023) emphasized the importance of feature selection methods in prediction models for eliminating unnecessary information, reducing computational costs during model training, and improving prediction performance. They underscored the necessity of using these methods and, within the scope of their study, proposed coefficient of variation (CV)-based feature selection approaches for stock prediction. The coefficient of variation, also known as relative standard deviation, is a statistical measure of data distribution. Therefore, to determine the importance of features considered in stock prediction, three different feature selection approaches based on this metric were proposed:

• *K-means algorithm:* The CV values calculated for each feature were subjected to k-means clustering, and the cluster with the largest size was selected. Consequently, the features corresponding to the CV values within the largest cluster were chosen.
• *Median range:* The median of the CV values calculated for each feature was computed. Then, a range was defined around the median value, and the features corresponding to the CV values within this range were selected.
• *Top-m:* The CV values calculated for each feature were sorted in descending order, and the top m features with the highest CV values were selected based on a predefined value of m.

To evaluate the proposed feature selection approaches, BPNN, LSTM, GRU, and CNN were used to predict the opening and closing prices of nine indices and stocks. Additionally, comparisons were made with five different feature selection approaches. The experiments and comparisons revealed that the proposed CV-based feature selection approaches improved the model’s prediction performance. However, it was noted that there were various challenges associated with using CV-based feature selection approaches, such as determining the value of m for the top-m approach and defining the median range to limit the number of features.

Beniwal, Singh & Kumar (2024) pointed out that existing studies on stock index forecasting in the literature generally focus on predicting the next day’s price. To address this gap in the literature, they investigated the impact of deep learning models on long-term (up to a year) stock index forecasting. In this regard, the performance of DNN, RNN, LSTM, CNN, GRU, and BiLSTM deep learning models for long-term price prediction of five major global stock indices such as Nifty, DJIA, DAX, NI225, and SSE was evaluated using RMSE and MAPE metrics. The findings demonstrate that LSTM performs well in long-term price prediction, and Bi-LSTM outperforms other models. Additionally, it was observed that the CNN model tends to overfit the training data and performs poorly on the test data. Based on the obtained findings, it was indicated that future research will explore different approaches to enhance the effectiveness of deep learning models in stock price prediction. These approaches include examining model performance using different data frequencies such as 1 min, 5 min, 15 min, *etc*., optimizing hyperparameters with evolutionary algorithms, and constructing hybrid prediction models using deep learning methods.

Wang, Liu & Jiang (2024) conducted a detailed investigation of stock price prediction studies in the literature. They observed that existing studies generally focus on point-value stock price prediction. However, considering that stock prices can exhibit complex trends in the real world, they suggested that interval-value prediction would be more suitable for this problem than point-value prediction. In this regard, they proposed an interval-valued decomposition integration model to predict stock indices’ high and low prices. The proposed model consists of three main modules: interval multiscale decomposition, multifactor analysis, and nonlinear integrated prediction based on intelligent optimization. Firstly, stock prices were decomposed into interval trends and residuals in the interval multiscale decomposition stage to conduct a more detailed analysis. The interval variational mode decomposition with feedback mechanism (FIVMD) method was employed for this decomposition process. Then, multifactor analysis was performed to examine the influence of external features on interval trends and residuals and identify important factors. These factors include fundamental factors such as global equity markets, gold market, foreign exchange market, carbon trading market, crude oil market and technical indicators affecting stock prices. The importance of all these external factors in stock price prediction, in other words, determining significant external factors, was investigated using the LightGBM method. In the final stage of the multifactor analysis module, dimensionality reduction was performed by applying the autoencoder method to the identified features, aiming to minimize computational complexity during model training. Finally, in the nonlinear integrated prediction based on an intelligent optimization module, interval trend, and residual series were separately predicted using the LSTM method optimized with the whale optimization algorithm (WOA). The proposed model was applied to the Shenzhen Stock Exchange Index (SZI), the Shanghai Stock Exchange Index (SSEC), and the China Securities 100 (CSI 100). It was compared with nine different models to investigate the impact of each module in the proposed prediction framework. The results indicate that the proposed model outperforms others and provides reliable stock price prediction. For future research, they plan to conduct investor sentiment analysis to explore factors affecting stock prices in detail. Additionally, they aim to select more complex models to enhance the existing prediction framework and increase prediction accuracy.

Akşehir & Kılıç (2024b), in another study, highlighted that time series data, especially financial time series, contain high levels of noise. They emphasized that the chaotic nature of financial time series, influenced by factors such as investor expectations, company policies, and political events, is the main reason for this noise. Upon examining the literature, they observed various approaches such as wavelet transform, Fourier transform, and mode decomposition being applied to remove noise from financial time series. Still, these approaches were not effectively eliminating the noise. To address this gap in the literature, they proposed the two-level entropy ratio-based CEEMDAN (2LE-CEEMDAN) approach for noise reduction. To evaluate the effectiveness of this denoising approach, they constructed a 2LE-CEEMDAN-LSTM-SVR prediction model and predicted the next day’s closing prices of the S&P 500, DAX, DJI, and SSE indices. This proposed prediction model consists of five stages: first decomposition, second decomposition, forecasting of second decomposition, forecasting of first decomposition, and ensemble unit. Initially, the CEEMDAN method was applied to the closing prices of stock indices in the first decomposition stage, and the obtained IMFs were classified as noiseless and high-frequency based on two different entropy metrics. Then, in the second decomposition stage, the CEEMDAN approach was applied again to the components classified as high-frequency in the previous stage, and the same classification process was repeated. Thus, noise, or high-frequency components, were effectively removed through these two stages. The obtained noiseless components were then fed into LSTM and SVR models. The prediction results obtained in the third and fourth stages were hierarchically combined to obtain the model’s final result. The performance of the proposed prediction model was compared with prediction models that used denoising approaches and those that did not. In both cases, it was observed that the proposed prediction model outperformed the other models, demonstrating the effectiveness of the proposed 2LE-CEEMDAN denoising approach. Akşehir & Kılıç (2024a) in another study, applied the relatively underutilized improved CEEMDAN approach in stock forecasting to reduce noise in financial time series. They observed that most proposed forecasting models in the literature train sub-series obtained through decomposition with the same prediction model despite the distinct characteristics of these sub-series, suggesting the need for individualized models for each sub-series. In response, they proposed a new hybrid forecasting model named ICE2DE-MDL based on decomposition-deep learning. They utilized ICE2DE-MDL to predict both stock market indices (CAC 40, FTSE 100, KOSPI, NASDAQ 100, NIKKEI, SET, S&P 500, SSE) and individual stocks (Xinning Logistics, Zhongke Electric, CITIC Securities) closing prices. They evaluated the performance of ICE2DE-MDL using statistical metrics and compared it with four different forecasting models from recent literature. The evaluation indicated that the ICE2DE-MDL outperformed other models across all datasets, demonstrating superior predictive performance. This underscores the effectiveness of the ICEEMDAN approach in reducing noise in financial time series and the beneficial impact of training each decomposed sub-series with its appropriate prediction model.

An et al. (2024) aimed to forecast stock prices and proposed a new hybrid prediction model, HFSLSMR-LSTM. This model comprises hierarchical feature selection with local shuffling (HFSLS) and models reweighting (MR) based on LSTM methods. They evaluated the HFSLSMR-LSTM model’s performance by predicting stock market indices and individual stock prices. To do so, they obtained approximately four years of closing price data from YahooFinance for eight different stock market indices (SSE, SSE 50, CSI 500, SZSE, CSI 300, SZSE 100, HSI, IXIC) and 30 stocks listed on the DJIA exchange. They then computed 158 technical indicators for each dataset to create feature sets, applying their proposed hierarchical feature selection approach to these sets. Finally, they conducted price forecasting using the LSTM method. To evaluate the performance of the proposed HFSLSMR-LSTM model, they utilized various statistical metrics (MSE, RMSE, MAE, MAPE, R-square) and compared them against six different forecasting models. Their findings and comparisons indicated that the HFSLSMR-LSTM prediction model is effective for financial time series characterized by multiple influencing factors and high volatility. Moreover, they demonstrated that the proposed model outperformed the six comparison models (DoubleEnsemble, Informer, GRU, LSTM, Bi-LSTM, Bi-GRU).

**Table 4:** Detail of stock prediction studies.

**Studies**	**Tasks**	**Stock item**	**Features**	**Feature selection**	**Feature extraction**	**Denoising**	**Train-Test approach**	**Prediction method**	**Performance metrics**
Chen & Zhou (2020)	Stock Price	China Construction bank, CSI 300 stock	Historical Price Data + Technical Indicators	Genetic algorithm	–	–	Traditional approach	LSTM	MSE
Chen, Zhang & Lou (2020)	Stock Index Price	S&P 500, Russell 2000, NASDAQ, DJI	Historical Price Data + Technical Indicators + Historical Data of Google Index + Natural Resources Prices	Pearson correlation analysis	–	–	Traditional approach	MLP + BiLSTM + Attention	MAE, MSE, *R*^2^, EVS, MSLE, MedAE
Kilimci & Duvar (2020)	Stock Trend	BIST 100 Bank Stocks	News + Forum Posts	–	–	–	Traditional approach	RNN, LSTM, CNN, Word Embedding Techniques (Word2Vec, GloVe, FastTex)	Accuracy
Liu & Long (2020)	Stock Index Price	S&P 500, DJI, CMSB	Decomposed Series with EWT	–	–	–	Traditional approach	LSTM Optimized with PSO	MAE, MAPE, RMSE, SDE
Lin et al. (2021)	Stock Trend	China Stocks	Historical Price Data + Technical Indicators	–	–	–	Traditional approach	LSTM, LR, SVM, RF, K-NN, GBDT	Accuracy, F-Score, Financial Evaluation
Ji, Liew & Yang (2021)	Stock Index Price	ASM, DJI, IXIC, N225, HSI	Historical Price Data	–	–	–	Traditional approach	LSTM Optimized with IPSO	MAE, MAPE, RMSE, *R*^2^
Althelaya, Mohammed & El-Alfy (2021)	Stock Index Price	S&P 500	Decomposed Series with Multiresolution Analysis	–	–	–	Traditional approach	LSTM	MAE, MAPE, RMSE, *R*^2^
Song, Baek & Kim (2021)	Stock Index Price	S&P 500, KOSPI, SSE	Historical Price Data	–	–	Padding-Based Fourier Transform	Traditional approach	LSTM, GRU, RNN	MAE, MAPE, RMSE
Wang et al. (2021)	Stock Index Price	S&P 500, NYSE, NASDAQ, DJI, FTSE 100, Nikkei 225, SSE	Historical Price Data	–	–	–	Traditional approach	Reservoir Computing	MAE, MAPE, RMSE, *R*^2^, MSE
Peng, Chen & Li (2021)	Stock Trend	US Stocks	Historical Price Data	Multiresolution analysis	–	Wavelet Transform	Traditional approach	DNN	Accuracy, F-Score
Gite et al. (2021)	Stock Price	Indian Stocks	Historical Price Data + News	–	–	–	Traditional approach	LSTM, LSTM-CNN	Accuracy
Ko & Chang (2021)	Stock Price	Taiwan Stocks	Historical Price Data + News + Forum Posts	–	–	–	Traditional approach	LSTM	RMSE
Yadav, Yadav & Saini (2022)	Stock Price	New York Stocks	Historical Price Data	–	–	–	Traditional approach	FastRNN, FastRNN-CNN- BiLSTM	RMSE
Tao et al. (2022)	Stock Price	China Stocks	Historical Price Data	–	ConvLSTM, GCN	–	Traditional approach	ConvLSTM-GCN- Dense	MSE, MAE, MAPE
Wang et al. (2022)	Stock Index Price	S&P 500, HSI, Nikkei 225	Historical price data	–	–	–	Traditional approach	Transformer	MAE, MSE, MAPE, Financial Evaluation
Lv et al. (2022)	Stock Index Price	S&P 500, HSI, Nikkei 225, DJI, SZ, SH	Decomposed Series	–	Deep Autoencoder	CEEMDAN	Sliding Window approach	LSTM	RMSE, MAE, NMSE, DC
Shah et al. (2022)	Stock Index Price	Nifty 50	Historical Price Data + Technical Indicators + Foreign Indices + Commodity Price + Exchange Rate	–	CNN	–	Traditional approach	LSTM	Accuracy, F-Score, MAPE, MSE, RMSE, *R*^2^, Financial Evaluation
Lawi, Mesra & Amir (2022)	Stock Price	GOOGL, AMZN, BLL, QCOM	Historical Price Data	–	–	–	Traditional approach	LSTM, GRU	MAPE, RMSPE, RMDPE
Chen, Wu & Wu (2022)	Stock Price	China’s Commercial Bank Stocks	Historical Price Data	–	–	–	Traditional approach	KD-LSTM	MAE, MSE, *R*^2^
Albahli et al. (2022)	Stock Trend	S&P 500 Stocks	Historical Price Data + Technical Indicators	–	–	–	Traditional approach	1D-DenseNet	MAE, RMSE, MAPE, AMAPE, PCT
Gupta, Bhattacharjee & Bishnu (2022)	Stock Index Price	Nifty 50	Historical Price Data	–	–	–	Traditional approach	GRU	RMSE, MAE, MAPE
Polamuri, Srinivas & Mohan (2022)	Stock Price	Stocks in NSE	Historical Price Data	XGBoost	Autoencoder	–	Traditional approach	GAN	MAE, MSE, Correlation
Akşehir & Kiliç (2022)	Stock Trading Signal	Stocks in Dow30	Historical Price Data + Technical Indicators + Gold Price + Oil Price	Correlation- based approach	–	–	Traditional approach, Sliding Window approach	2D-CNN	Recall, Precision, F-Score, Accuracy
He, Siu & Si (2023)	Stock Trend	Stocks in NYSE and NASDAQ	Historical Price Data	–	–	–	Traditional approach	IDTLA	Accuracy, Matthews Correlation Coefficient (MCC)
Chen & Zhu (2023)	Stock Trading Signal	Stocks in US and TR, ETF	Historical Price Data + Technical Indicators	–	–	–	Sliding Window approach	PLR-CNN-LSTM	Recall, Precision, F-Score, Financial Evaluation
Mu et al. (2023)	Stock Price	China Stocks	Historical Price Data + Textual Data	–	–	–	Traditional approach	MS-SSA-LSTM	MAE, MAPE, RMSE, *R*^2^
Zhang, Ye & Lai (2023)	Stock Index Price	CSI 300	Historical Price Data	–	CNN	–	Traditional approach	CNN-BiLSTM- Attention	MAPE, RMSE, *R*^2^
Cagliero, Fior & Garza (2023)	Stock Trend	Stocks in S&P 500 and MIB 40	Historical Price Data + Candlestick Data	–	–	–	Traditional approach	Pattern Recognition + (MLP, SVM, LSTM)	Financial Evaluation
Kim et al. (2023)	Stock Index Trend	KOSPI 200	Historical Price Data + Technical Indicators	–	–	–	Traditional approach	CNN+Grad-CAM	Financial Evaluation
Qi, Ren & Su (2023)	Stock Index Price	S&P 500, CSI 300	Decomposed Series	–	–	CEEMDAN- Wavelet Transform	Traditional approach	GRU based on CEEMDA-Wavelet	MSE, MAE, RMSE, *R*^2^
Yao, Zhang & Zhao (2023)	Stock Index Price	SSEC, DJI, FTSE 100, FCHI, Nikkei 225, IRTS, STI	Decomposed Series	–	–	–	Traditional approach	MEMD-TCN	RMSE, MAPE, DA, MASE
Zhao & Yang (2023)	Stock Index Trend	HSI	Historical Price Data + Technical Indicators + Textual Data	–	Denoising autoencoder	–	Sliding Window approach	SA-DLSTM	MAPE, Financial evaluation
Jarrah & Derbali (2023)	Stock Price	Stocks in SSI	Historical Price Data	–	–	Exponential smoothing	Traditional approach	LSTM	MAPE, MSE, MAE, RMSE
Wang & Zhu (2023b)	Stock Index Trend	SSE 50, CSI 300	Historical Price Data + Textual Data	–	CNN	–	Traditional approach	Dual Classifier Coupling (CNN-SVM)	Accuracy, Recall, Precision, F-Score, AUC
Md et al. (2023)	Stock Price	Samsung Stock	Historical Price Data	–	–	–	Traditional approach	LSTM	MAPE, RMSE, *R*^2^
Zaheer et al. (2023)	Stock Index Price	Shanghai Composite Index	Historical Price Data	–	–	–	Traditional approach	CNN, LSTM, RNN	MAE, RMSE, *R*^2^
Wang & Zhu (2023a)	Stock Index Price	SSE, SZSE, NYSE	Historical Price Data + Non-Basic Factors	Multi-factor analysis	–	–	Traditional approach	LSTM Optimized with GA	MAPE, MSPE, MAE, RMSE
Dahal et al. (2023)	Stock Price	Stocks in Taiwan Stock Exchange	Historical Price Data + News + Forum Posts	–	–	–	Traditional approach	LSTM	RMSE
Yu et al. (2023)	Stock Index RV	S&P 500, SSEC, FTSE	Decomposed Series	–	–	–	Traditional approach	GVMD-Q-DBN- LSTM-GRU	MAE, MSE, HMAE, HMSE
Albahli et al. (2023)	Stock Trend	S&P 500 Stocks	Historical Price Data + Technical Indicators	–	–	–	Traditional approach	DenseNet-41	MAE, RMSE, MAPE, AMAPE, PCT
Fang et al. (2023)	Stock Index Trend	S&P 500, CSI 300, SSE 180	Historical Price Data	–	–	–	Traditional approach	LSTM-BN	Accuracy, Financial Evaluation
Song & Choi (2023)	Stock Index Price	S&P 500, DJI, DAX	Historical Price Data	–	–	–	Traditional approach	CNN-LSTM, GRU-CNN, Ensemble Model (LSTM-GRU-RNN)	MSE, MAE
Cui et al. (2023)	Stock Index Price	S&P 500, SSCI, FTSE, STI	Decomposed Series	–	Convolutioal Block Attention Module	Multi-Channel VMD	Sliding Window approach	McVCsB	MSE, MAE, HMSE, HMAE
Chaudhari & Thakkar (2023)	Stock/Stock Index Price/Trend	Seven Stock Items	Historical Price Data + Technical Indicators	CV-based approaches	–	–	Traditional approach	BPNN, LSTM, GRU, CNN	DA, Sensitivity, Specificity, F-Score, MAE, MSE, *R*^2^, Kappa
Beniwal, Singh & Kumar (2024)	Stock Index Price	Nifty, DJI, DAX, Nikkei 225, SSE	Historical Price Data	–	–	–	Traditional approach	DNN, RNN, LSTM, CNN, GRU, BiLSTM	RMSE, MAPE
Wang, Liu & Jiang (2024)	Stock Index Price	SZI, SSEC, CSI 100	Decomposed Series	Multi-factor analysis with LightGBM	–	–	Traditional approach	LSTM optimized with WOA	RMSE, MAE, MAPE
Akşehir & Kılıç (2024b)	Stock Index Price	S&P 500, DAX, DJI, SSE	Decomposed Series	–	–	2LE-CEEMDAN	Traditional approach	2LE-CEEMDAN- LSTM-SVR	RMSE, MAE, MAPE, *R*^2^
Akşehir & Kılıç (2024a)	Stock Index Price Stock Price	CAC 40, FTSE 100, KOSPI, NASDAQ 100, NIKKEI, SET, S&P 500, SSE, Xinning logistics, Zhongke electric, CITIC securities	Decomposed Series	–	–	ICEEMDAN and Entropy	Traditional approach	ICE2DE-MDL	RMSE, MAE, MAPE, *R*^2^
An et al. (2024)	Stock Index Price Stock Price	SSE, SSE 50, CSI 500, SZSE, CSI 300, SZSE 100, HSI, IXIC, and DJIA Stocks	Historical Price Data + Technical Indicators	HFSLSMR	–	–	Traditional approach	HFSLSMR-LSTM	RMSE, MSE, MAE, MAPE, *R*^2^

**Table 5:** Sources of datasets used in studies.

**Studies**	**Dataset Source**
Chen & Zhou (2020)	https://www.joinquant.com
Chen, Zhang & Lou (2020), Ji, Liew & Yang (2021), Althelaya, Mohammed & El-Alfy (2021), Lawi, Mesra & Amir (2022), Albahli et al. (2022), Gupta, Bhattacharjee & Bishnu (2022), Akşehir & Kiliç (2022), Md et al. (2023), Zaheer et al. (2023), Albahli et al. (2023), Chaudhari & Thakkar (2023), Akşehir & Kılıç (2024a), Akşehir & Kılıç (2024b), An et al. (2024)	Yahoo Finance
Kilimci & Duvar (2020)	Twitter, Bigpara, Public Disclosure Platform (KAP), and Mynet Finans
Liu & Long (2020), Wang et al. (2021), Wang et al. (2022), Shah et al. (2022), Chen & Zhu (2023), Zhang, Ye & Lai (2023), Cagliero, Fior & Garza (2023), Kim et al. (2023), Qi, Ren & Su (2023), Yu et al. (2023), Fang et al. (2023), Song & Choi (2023), Cui et al. (2023), Beniwal, Singh & Kumar (2024), Wang, Liu & Jiang (2024)	Unspecified
Lin et al. (2021)	CCER, a local data provider of China
Song, Baek & Kim (2021)	Yahoo Finance and https://www.investing.com
Peng, Chen & Li (2021)	The center for research in security prices (CRSP)
Gite et al. (2021)	Yahoo Finance and Pulse
Ko & Chang (2021)	PTT platform
Yadav, Yadav & Saini (2022)	https://github.com/MilindYadav-97/Hybrid_FastRNN-for-stock-predictions
Tao et al. (2022), Lv et al. (2022)	https://tushare.pro/
Chen, Wu & Wu (2022)	Wind database
Polamuri, Srinivas & Mohan (2022)	https://www.nseindia.com/
He, Siu & Si (2023)	https://github.com/yumoxu/stocknet-dataset https://github.com/fulifeng/Adv-ALSTM/tree/master/data/kdd17/ourpped https://www.cis.fordham.edu/wisdm/dataset.php
Mu et al. (2023)	Ruisi Financial database and East Money forum posts
Yao, Zhang & Zhao (2023)	China Stock Market & Accounting Research Database
Zhao & Yang (2023)	Use the web crawler to download the textual contents
Jarrah & Derbali (2023)	https://www.investing.com
Wang & Zhu (2023b)	East money.com Stock Index Forum
Wang & Zhu (2023a)	Oriental Fortune Choice database
Dahal et al. (2023)	Yahoo Finance, Reddit World News Channel and Finviz

The information regarding the 49 summarized studies is provided in Table 4, including the problem addressed, the specific stock item focused on, the variables utilized as input features, the preferred methods of feature selection/extraction and denoising pre-processing steps are applied, the train-test approach used, the preferred deep learning approach as the prediction method, and details on the metrics used to evaluate prediction performance. Additionally, information on where or from which sources the datasets used in the studies were obtained is provided in Table 5. Upon review, it was observed that the majority of the studies obtained their datasets through Yahoo Finance. However, it was also found that most studies did not provide any information on the source of the datasets used. One of the studies reviewed observed that a GitHub link for the dataset used was provided.

### RQ1: Which deep learning methods are preferred in stock prediction models?

Based on the examined studies, it is observed that the LSTM model, which has proven its success on time series data, is widely used in stock market prediction problems. Additionally, some studies have also employed hyperparameter optimization methods such as WOA, PSO, IPSO, and genetic algorithms in conjunction with the LSTM method. These studies have demonstrated that focusing on model development techniques like hyperparameter optimization, in addition to deep learning methods, can enhance the model’s prediction performance. Moreover, recurrent neural network types such as GRU, BiGRU, BiLSTM, and CNN are also frequently preferred.

Literature reviews have revealed that approaches utilizing transformer models and hybrid prediction models incorporating methods such as DenseNet and ConvLSTM have not yet been applied in the financial time series prediction field. Therefore, determining whether these methods can be effectively utilized in stock prediction problems remains an open question. Additionally, it has been reported that hybrid approaches combining multiple methods tend to yield more successful results than solely relying on artificial intelligence techniques (Zhang, Sjarif & Ibrahim, 2022; Chopra & Sharma, 2021; Kanwal et al., 2022). In this regard, it is considered beneficial for researchers to focus on developing hybrid approaches that would exhibit superior performance in future studies.

### RQ2: Which input features do the proposed prediction models focus on?

The data type used in stock market prediction significantly influences the model’s performance. If the factors influencing the commodity whose price/direction is to be predicted are well analyzed, and these data are collected from various sources and combined, the prediction performance will likely improve. However, the market’s susceptibility to many factors, its random behavior, and its complexity make this analysis challenging and limit the success it achieves (Kumbure et al., 2022). When examining stock market prediction studies, the data used in prediction models can be categorized into five main headings: price, technical indicators, fundamental indicators, and alternative/textual data, along with macroeconomic variables (Rouf et al., 2021; Kumbure et al., 2022):

• *Price Data:* Daily, monthly, hourly, or minute-based data showing the historical price data of commodities. This price data includes the commodity’s highest, lowest, opening, closing, and volume.
• *Technical Indicator Data:* Based on the philosophy that price is the indicator of everything, the technical analysis aims to predict the future direction of a commodity by assuming that any factor that can affect the market price of a commodity directly affects its price. This analysis is carried out through technical indicators. There are many technical indicators in the market, and these indicators are categorized into four headings based on the information they provide: momentum, trend, volatility, and strength indicators.
• *Fundamental Indicator Data:* Fundamental data includes information about the company to which the commodity is linked. These data encompass information representing the company’s situation, such as its income and expenditure status, financial reports, and growth potential.
• *Macroeconomic Variables:* Exchange rates and other commodity prices (such as oil, gold, *etc*.) are evaluated under this category.
• *Alternative/Textual Data:* Social media posts, financial news, and comments are included in this category.

It was observed that the majority of the examined studies have utilized historical price data of stocks/indexes as input variables for the prediction model. Some studies have also considered calculated technical indicator values derived from historical price data, referred to as technical analysis. Additionally, in particular studies, it has been suggested that comments or news about the stock/index made on social media may affect the predicted asset’s behavior, leading to the collection of textual data about the relevant asset and subsequent sentiment analysis. However, collecting such textual data and performing sentiment analysis on unstructured data is acknowledged to be a challenging process. Therefore, these textual data are less preferred than historical price data and technical indicators in studies. Similarly, macroeconomic indicators, like textual data, are rarely chosen due to the difficulty in collecting them, unlike easily accessible historical price data. Consequently, enhancing model performance by utilizing macroeconomic indicators and developing effective sentiment analysis methods is crucial. Researchers are encouraged to prioritize these aspects in their studies for improved outcomes.

### RQ3: How often are feature extraction-selection methods used in prediction models, and what is their impact on model performance?

Another important part for the proposed prediction models is feature selection and extraction methods. Various factors that are likely to influence the financial asset to be predicted are used to form the initial set of input variables. However, based on research conducted and findings obtained, although these variables are presumed to affect the financial asset directly, they may not necessarily have significance for the prediction model in practice. Therefore, it is necessary to apply a feature selection approach to these initial input variables to identify and remove unnecessary/insignificant variables from the input variable set. Thus, both the computation cost during model training will decrease, and the potential for the model to make incorrect decisions will be mitigated to some extent. In this context, a study was conducted on whether feature selection approaches were used in the examined studies, and it was observed that these techniques were utilized in approximately 17% of the studies. Genetic algorithms, Pearson correlation analysis, XGBoost, LightGBM, and multi-factor analysis were observed as the feature selection methods used in these studies. These proposed feature selection approaches have been proven to impact the model performance positively. Furthermore, using feature extraction approaches also directly affects the model’s prediction performance. However, when examining the studies, it was observed that deep learning models leverage feature extraction inherent in their architectural structure instead of directly using a feature selection approach. Therefore, when filling in the section regarding feature selection approaches in Table 4, consideration was given to whether an external feature extraction approach was used. In this regard, CNN and autoencoder methods were generally used. These studies on feature selection/extraction methods used in stock prediction problems indicate that these methods should be given more importance, especially in complex problems like financial time series. Researchers need to focus on more detailed research on feature selection/extraction approaches, which are critical for improving the prediction performance of the models and reducing unnecessary information load.

### RQ4: What methods are proposed for denoising stock data, and how often are they preferred?

Due to the non-stationary and non-linear structures of financial market data, sharp/sudden changes in price behaviors are common. It has been observed that approximately 74% of the examined studies do not focus on detecting sudden change points in these data or eliminating noise that causes sudden changes. Therefore, it has been observed that directly applying prediction models to these data leads to incorrect decisions. Hence, applying a denoising approach to financial time series data during the data preprocessing stage is necessary to smooth out these sudden changes and train prediction models on denoised data. This will prevent investors from making incorrect decisions and thus enable them to achieve higher returns. In the examined studies, various decomposition methods such as Fourier transform, wavelet transform, CEEMDAN, 2LE-CEEMDAN and deep learning techniques such as denoising autoencoders have been attempted to address the noise problem causing incorrect decisions. As a result of the reviews, it has been observed that instead of directly using noisy data in prediction models, further improvement in model performance can be achieved by first removing noise from the data. Therefore, for future studies, researchers may find it beneficial to explore noise removal techniques in the literature and evaluate their impact on model performance by applying them to financial time series data.

### RQ5: Which approach, sliding window or traditional train-test splitting, is more commonly preferred in prediction models?

It has been observed that the traditional train-test approach is generally preferred in the studies. According to the reviews, it was determined that only five of the 49 examined studies used the sliding window method. The traditional train-test approach typically involves partitioning the dataset based on a specific period to evaluate the model at different time intervals during the training and testing phases. This approach is widely used in the literature and is considered reliable by researchers. In contrast, the sliding window approach, which allows for more dynamic processing of time series data and enables the model to be trained with more up-to-date data at different time intervals, is less preferred in model training. However, this approach is important, especially for capturing trends and patterns in financial time series data that change over time. Therefore, it is necessary for the sliding window method to be used more frequently in future studies, and the advantages of this method should be explored in more detail.

### RQ6: What metrics are used to evaluate the performance of models?

In evaluating the performance of stock market prediction models, it is essential to pay attention not only to commonly used statistical metrics in artificial intelligence techniques but also to financial evaluation metrics. Statistical metrics such as MSE, MAE, RMSE, MAPE, *R*^2^, accuracy, precision, recall, and F1 score provide a general overview of the models’ performance. However, to fully assess the success of stock market prediction models, it is necessary to consider financial evaluation metrics such as average return, Sharpe ratio, and trading simulations. Upon reviewing the literature, it is observed that the majority of studies evaluate the model’s prediction performance using only statistical metrics, while 13% of the reviewed studies consider both types of metrics. In future research, emphasis should be placed on the necessity of using both statistical and financial metrics together to understand the effectiveness of stock market prediction models fully. This approach will allow for a more comprehensive evaluation of the models’ success under real-world conditions.

## Conclusions and Research Directions

There are a considerable number of stock forecasting studies in the literature, and this study focuses on deep learning-based stock forecasting studies between 2020 and 2024, conducting a systematic literature review. In this context, literature searches were conducted on the IEEE Xplore, Scopus, and Web of Science databases, and 49 studies to be examined within the scope of this review were identified. The selected studies were thoroughly examined in terms of the deep learning method, input features, feature selection-extraction methods, denoising approaches, train-test approaches, and performance metrics. As a result of these reviews:

• When evaluated by year, it is observed that the number of open-access articles has been increasing.
• It has been observed that deep learning methods, especially LSTM, are frequently preferred in stock forecasting models. This can be attributed to LSTM’s ability to model long-term dependencies effectively and capture complexity in time series data.
• When examining the proposed forecasting models, it was observed that hybrid models outperform single models.
• It is noteworthy that historical price data is generally used in forecasting models. This can be attributed to the ease of accessibility of these data and the assumption that all factors affecting the stock item are reflected in price data.
• Notably, very few of the proposed forecasting models utilize feature selection and/or extraction approaches.
• The majority of forecasting models lack noise reduction in stock data. However, stock data contains high noise levels due to its characteristic nature, and this directly affects prediction performance negatively.
• While traditional train-test splits are generally preferred during model training, the use of the sliding window approach was observed in very few studies.
• In the evaluation of proposed forecasting models, statistical metrics are frequently utilized, and in some studies, both statistical and financial metrics are employed.

As a result of the evaluations, open problems have been identified in the following issues, and recommendations have been made:

• When evaluating the effectiveness of deep learning methods in stock forecasting models, it is important to adapt successful methods from the natural language processing field, such as the transformer method, to stock prediction problems and test their success.
• More effective use of feature selection approaches in stock forecasting problems can enhance the prediction success of models. Studies should be conducted to develop new hybrid feature selection approaches.
• It is observed in the literature that the mode decomposition methods applied to remove noise in stock data are generally single variable-based. The use of multi-variable-based denoising approaches can positively contribute to the stock prediction problem. Therefore, focus should be placed on research on multi-variable-based noise reduction approaches.
• In deep learning-based stock forecasting studies, a separate method for uncovering hidden patterns is not typically preferred. Therefore, developing external feature extraction methods is necessary to create more successful prediction models.
• It is considered insufficient only to use statistical metrics when evaluating the prediction performance of models. In addition to these metrics, conducting trading simulations to evaluate the practical performance of the model is important. Considering both types of metrics can demonstrate the success of the prediction model both theoretically and practically. Therefore, researchers are recommended to use these metrics together.
• In stock forecasting studies in the literature, it is observed that the dataset is generally divided into a 7:3 ratio for model training. However, this approach is thought to limit the model’s generalization ability. Therefore, a sliding window-based training-test approach for data with changing values over time is more appropriate. This method will allow the model to better adapt to changing market conditions over time. Therefore, researchers are advised to give more weight to the sliding window-based training-test approach.

## Additional Information and Declarations

### Data Availability

The following information was supplied regarding data availability: This is a literature review.
